# Biomass RNA for the Controlled Synthesis of Degradable Networks by Radical Polymerization

**DOI:** 10.1021/acsnano.3c08244

**Published:** 2023-10-18

**Authors:** Jaepil Jeong, So Young An, Xiaolei Hu, Yuqi Zhao, Rongguan Yin, Grzegorz Szczepaniak, Hironobu Murata, Subha R. Das, Krzysztof Matyjaszewski

**Affiliations:** † Department of Chemistry, Carnegie Mellon University, Pittsburgh, Pennsylvania 15213, United States; ‡ Center for Nucleic Acids Science & Technology, Carnegie Mellon University, Pittsburgh, Pennsylvania 15213, United States; § Department of Materials Science & Engineering, Carnegie Mellon University, Pittsburgh, Pennsylvania 15213, United States; ∥ University of Warsaw, Faculty of Chemistry, Pasteura 1, 02-093 Warsaw, Poland

**Keywords:** RNA, hydrogel, biomass, acylation, ATRP, RAFT, free radical polymerization

## Abstract

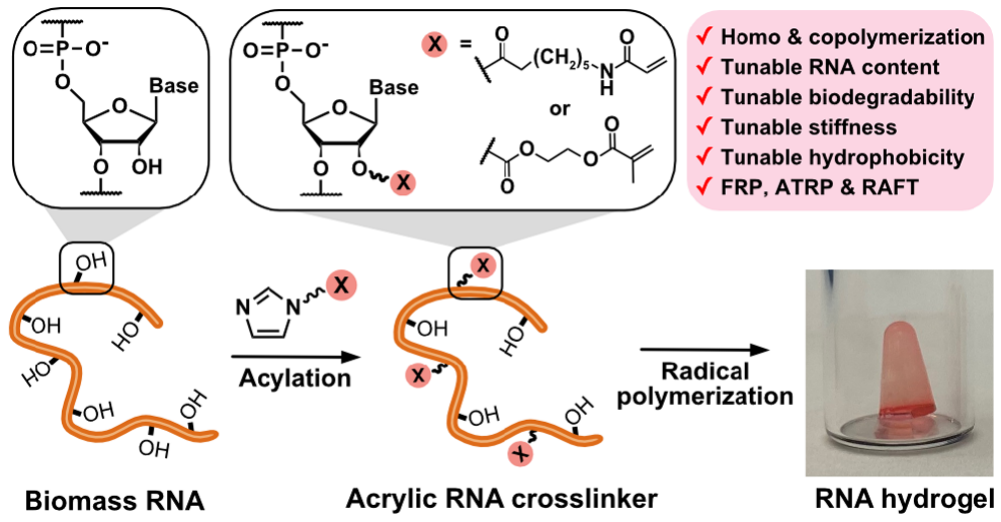

Nucleic acids extracted from biomass have emerged as sustainable and environmentally friendly building blocks for the fabrication of multifunctional materials. Until recently, the fabrication of biomass nucleic acid-based structures has been facilitated through simple crosslinking of biomass nucleic acids, which limits the possibility of material properties engineering. This study presents an approach to convert biomass RNA into an acrylic crosslinker through acyl imidazole chemistry. The number of acrylic moieties on RNA was engineered by varying the acylation conditions. The resulting RNA crosslinker can undergo radical copolymerization with various acrylic monomers, thereby offering a versatile route for creating materials with tunable properties (e.g., stiffness and hydrophobic characteristics). Further, reversible-deactivation radical polymerization methods, such as atom transfer radical polymerization (ATRP) and reversible addition–fragmentation chain transfer (RAFT), were also explored as additional approaches to engineer the hydrogel properties. The study also demonstrated the metallization of the biomass RNA-based material, thereby offering potential applications in enhancing electrical conductivity. Overall, this research expands the opportunities in biomass-based biomaterial fabrication, which allows tailored properties for diverse applications.

## Introduction

Nucleic acids have been widely utilized as versatile building blocks for the fabrication of multifunctional biomaterials.^1−3^ In addition to their intrinsic biological activity, molecular recognition and programmable self-assembly further enabled the use of nucleic acids for nanofabrication at the subnanometer precision.^4^ Over the past few decades, significant progress has broadened the application of nucleic acids as building materials. This expansion encompasses synthetic oligonucleotides,^5−7^ enzymatically amplified sequences,^8−11^ and nature-derived nucleic acids extracted from biomass.^12,13^

Among the various sources of nucleic acids, biomass extracts have received growing attention as building blocks, particularly for the synthesis of macroscopic structures. This is mainly because of the relatively low cost of biomass DNA and RNA compared with other types of nucleic acids. Additionally, these sustainable natural biopolymers could be extracted from any living organisms, thereby potentially reducing the use of petrochemicals.^14,15^ For example, since there are approximately 50 billion metric tons of biomass DNA on the Earth,^16^ replacement of the current annual production of commodity plastics would require converting only ∼0.7% of the biomass DNA on the Earth.^17^ Consequently, a variety of macroscopic 3D structures, such as commodity plastics,^14^ hydrogels,^15^ aerogels,^12^ and optoelectronic devices,^18^ have employed biomass DNA as the building blocks.

**Figure 1:**
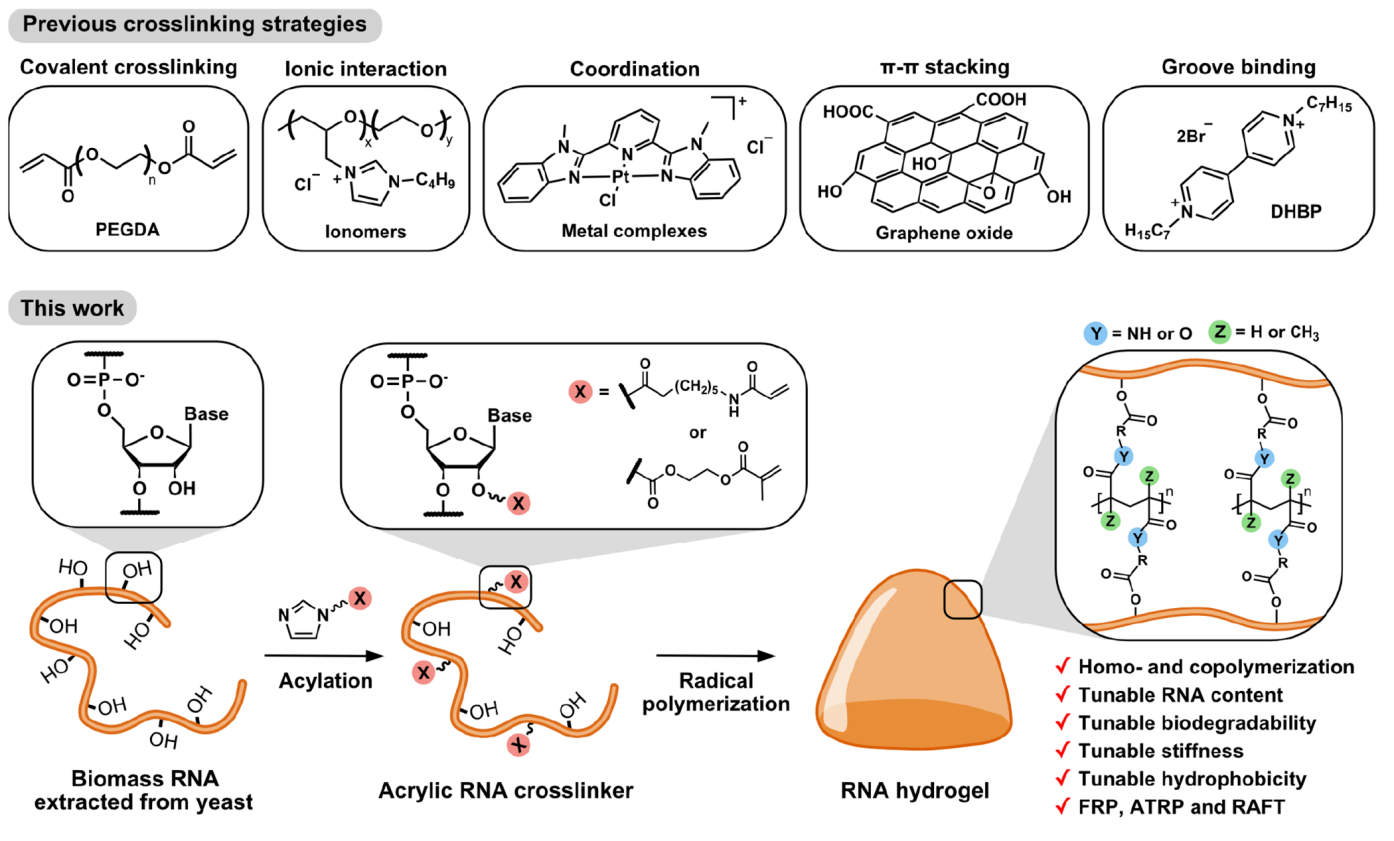
Transformation of biomass RNA into acrylic crosslinker and the fabrication of degradable networks by radical polymerization.

Until recently, direct crosslinking of biomass DNA has been the main approach for fabricating biomass DNA-based material (Figure 1, top). In one of the approaches, nitrogenous bases in DNA were covalently crosslinked through nucleophilic addition reactions using crosslinkers, such as poly(ethylene glycol) diacrylate (PEGDA)^15^ or ethylene glycol diglycidyl ether (EGDE).^19,20^ Additionally, electrostatic interactions,^14,21,22^ coordination,^23,24^ π–π stacking,^25−27^ and groove binding^18^ have been exploited. These noncovalent methods offer the advantage of reshaping and reprocessing the resulting material because of the weak and reversible nature of the interactions. Furthermore, these biomass DNA-based materials were metalized via treatment with metal precursors (e.g., HAuCl_4_), which enabled their use as catalysts.^20^ Nevertheless, the simple crosslinking of biomass DNA constrains the possibility of engineering material properties since incorporating functional molecules (e.g., synthetic polymers) is challenging.^28^

To address this challenge, we sought to use biomass RNA, which to the best of our knowledge has not been reported previously. We aimed to functionalize biomass RNA with polymerizable vinyl groups rather than simply crosslinking nucleic acids (Figure 1, bottom). This strategy for functionalization takes advantage of the 2′-hydroxyl groups in RNA to convert biomass RNA into acrylic crosslinkers, which can undergo subsequent polymerization through radical polymerization. The biomass RNA functionalization was leveraged through acyl imidazole chemistry: the covalent reaction between 2′-hydroxyl groups in RNA with acylating reagents, particularly acyl imidazole, to form 2′-O-adduct. Unlike other chemistries for nucleic acid modification, acyl imidazole chemistry has the advantage of using biologically relevant conditions with less toxic imidazole as a byproduct.^29^ Consequently, acyl imidazole chemistry has been extensively studied for the structural mapping of RNA (i.e., selective 2′-hydroxyl acylation analyzed by primer extension, SHAPE) in vitro^30^ and in vivo.^31^ We have recently demonstrated that RNA can be modified with acyl imidazole reagents to serve as an atom transfer radical polymerization (ATRP) initiator under the mild and biocompatible polymerization conditions.^32^ Additionally, it is important to note that during the radical polymerization process vinyl monomers could undergo copolymerization with two or more different types of monomers. As a result, a distinctive material emerges with contributing properties from each monomer, which leads to a tailored material for specific needs.^33,34^ Synthetic oligonucleotides may be equipped with polymerizable handles (e.g., methacrylamide or norbornene) and incorporated into polymeric networks through radical polymerizations^35−37^ or ring-opening metathesis polymerizations (ROMP).^38−40^ However, such methodology for nucleic acid modification can only be achieved by leveraging phosphoramidite chemistry during solid-phase synthesis, which is not applicable to biomass nucleic acids. Consequently, our strategy greatly broadens the scope of possible functionalities through radical (co)polymerization methods for biomass RNA-based 3D-structured materials. This expansion allows access to outstanding potential applicability in various fields, including 3D printing, healthcare, electronics, and catalysis.

## Results and Discussion

### Synthesis of Acrylamido RNA Crosslinkers under Different Acylation Conditions

**Figure 2:**
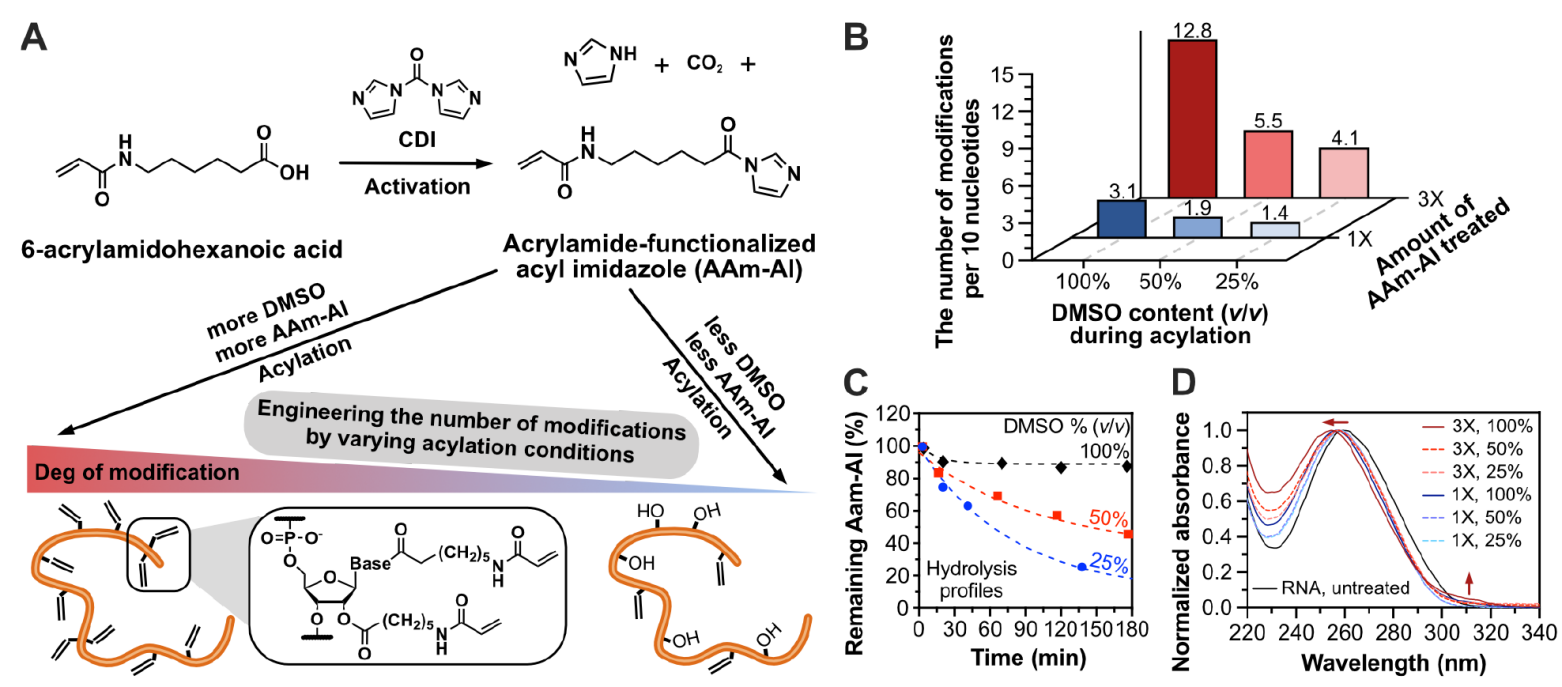
Synthesis of acylating reagent and acrylamido RNA crosslinker. (A) Synthesis of the **AAm-AI** reagent and treatment of biomass RNA with **AAm-AI** under different conditions to engineer the degree of modification. (B) The number of acrylamide modifications per 10 ribonucleotides. 1× and 3× **AAm-AI** refer to 1 and 3 equiv of **AAm-AI** compared with ribonucleotides, respectively. ^1^H NMR was used for the quantification (Figure S5). (C) Hydrolysis kinetics of the **AAm-AI** reagent under the different concentrations of DMSO in water on the basis of NMR peak analysis. The ^1^H NMR spectra are shown in Figures S6–S8. (D) UV–vis spectra of acrylamido RNA crosslinker synthesized under different conditions.

Acrylamide-functionalized acyl imidazole (**AAm-AI**) was synthesized as a model acylating reagent in the reaction of 6-acrylamidohexanoic acid with 1,1′-carbonyldiimidazole (CDI) in DMSO (Figure 2A). The formation of **AAm-AI** was confirmed by NMR spectroscopy (Figure S1). The freshly synthesized **AAm-AI** was added to commercially available biomass RNA extracted from the torula yeast. The resulting acrylamido RNA crosslinker was isolated by precipitation with isopropanol. We confirmed through 2D heteronuclear single-quantum coherence (HSQC) NMR analysis (^1^H–^13^C) of the biomass RNA before (Figure S2) and after (Figure S3A) **AAm-AI** treatment that the acrylamide linkers were successfully incorporated in the biomass RNA. The results of the proton diffusion-ordered spectroscopy (^1^H-DOSY) experiment also showed that the protons in the RNA and the acrylamido moieties have identical diffusion coefficients, thereby indicating that they were in the same molecule (Figure S3B). We further characterized the biomass RNA crosslinker using size exclusion chromatography equipped with multiangle light scattering (SEC-MALS) as illustrated in Figure S4 (*M*_n,MALS_ = 12 850, *Đ* = 1.18).

Next, we quantified the degree of **AAm-AI** modification of RNA. Importantly, previous studies have shown that the acylation yield can be influenced by the reaction conditions, including the amount of acylating reagent used or the ratio of cosolvent (e.g., DMSO) to water.^29^ Therefore, we synthesized acrylamido RNA crosslinkers under different acylation conditions (% DMSO v/v in water and the amount of **AAm-AI**), and the degree of acylation was determined using ^1^H NMR, according to the previously reported procedure (Figure S5).^32^ As shown in Figure 2B, conducting RNA modification with a higher concentration of DMSO in water or using a larger amount of **AAm-AI** resulted in an increased number of acrylamide incorporation in the RNA. It should be noted that up to superstoichiometric yield (12.8 acrylamide groups per 10 ribonucleotides) could be achieved when RNA was treated with 3 equiv (3×) of **AAm-AI** compared with ribonucleotide in 100% DMSO overnight. The enhanced acylation under high % DMSO is attributed to the slower hydrolysis of **AAm-AI** under such conditions (Figure 2C). During RNA acylation, hydroxyl groups in RNA compete with the surrounding water molecules because of the hydrolysis of acylating reagents in water. Consequently, less water for acylation could lead to a longer half-life of **AAm-AI** and an enhanced degree of modification, as confirmed by ^1^H NMR spectroscopy (Figures S6–S8). The RNA crosslinkers were further characterized by UV–vis, as shown in Figure 2D. The shift of the characteristic absorption peak of RNA at 260 nm, which corresponds to nucleobases, indicated that **AAm-AI** reacted with nitrogenous bases in addition to 2′-hydroxyl groups.^41^ Of note, the most significant shift (∼5 nm) was observed for the 3×, 100% DMSO condition (Figure 2D, red solid line) compared with other conditions (∼0.5–2 nm) because water shows stronger nucleophilicity to most acylating reagents than amine groups on RNA bases.^29^ These results suggest that the binding site and the number of acrylamido functional groups on the RNA crosslinker can be engineered by tuning the experimental conditions, including the acylation media or the amount of acylating reagent.

### Control over the Degradability of RNA Hydrogels by Tailoring the Degree of Acylation

**Figure 3:**
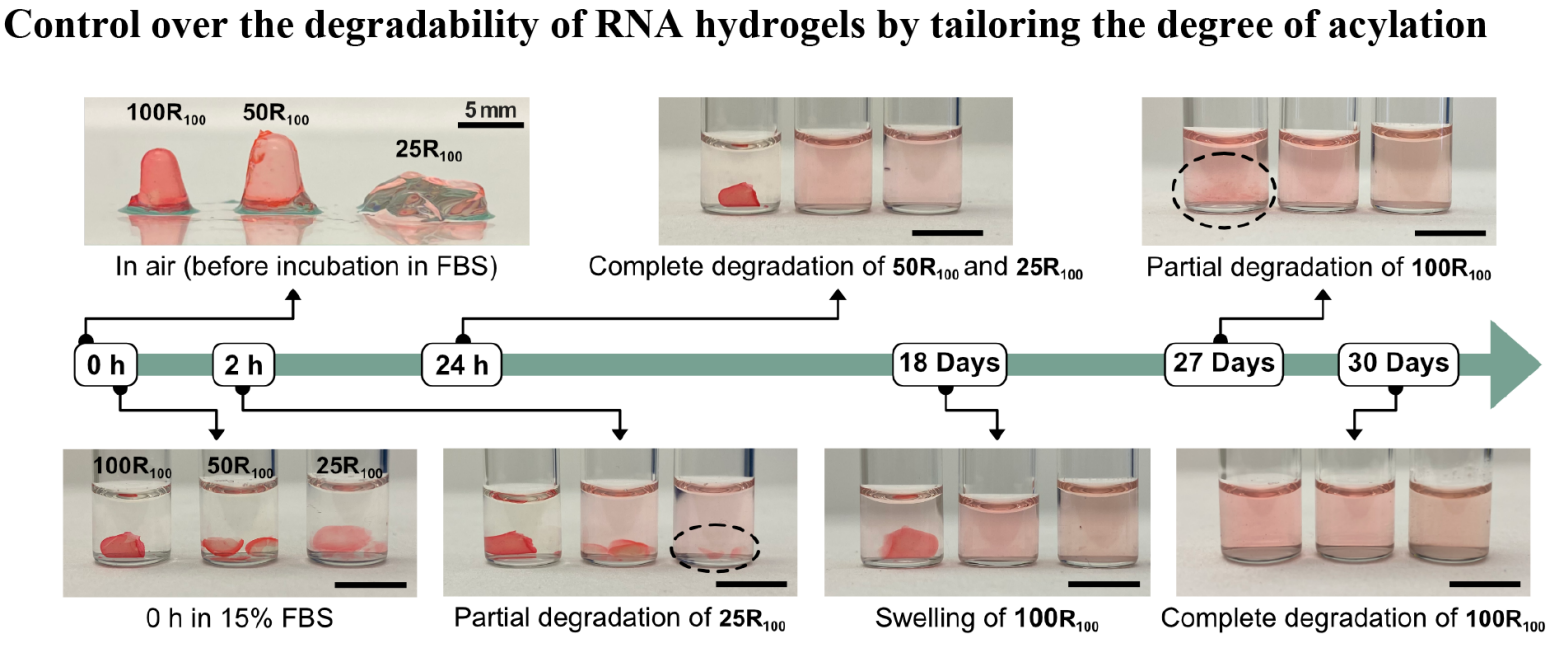
The effect of the degree of modification to the degradability of RNA hydrogels in 15% FBS. The **100R**_**100**_, **50R**_**100**_, and **25R**_**100**_ hydrogels were synthesized by homopolymerization of RNA crosslinker that was prepared under 100%, 50%, and 25% of DMSO in water (v/v), respectively. The RNA hydrogels were stained with the GelRed dye for easier visualization. Scale bars: 5 mm. A summary of the reaction conditions for the synthesis of the hydrogels is shown in Table S1.

With the acrylamido RNA crosslinkers in hand, we proceeded to test the capability of acrylamido residues in RNA to undergo polymerization via free radical polymerization (FRP). Three different RNA crosslinkers were synthesized in 100%, 50%, or 25% DMSO in water (v/v) using 3× of **AAm-AI** to ribonucleotide, respectively. Each RNA crosslinker was then homopolymerized by FRP using ammonium persulfate (APS) and tetramethylethylenediamine (TEMED) in water. After 5 min of polymerization at room temperature, the RNA hydrogels were stained with GelRed, a nucleic acid-intercalating dye, for visualization (Figure 3, left top). Notably, the RNA hydrogel (**25R**_**100**_), fabricated using the RNA crosslinker prepared in 25% DMSO (v/v), could not stand independently outside of water under the polymerization conditions tested. This can be attributed to the lower number of crosslinking points compared with RNA crosslinkers synthesized in 100% (**100R**_**100**_) or 50% (**50R**_**100**_) DMSO (v/v) (Figure 2B). Enhanced crosslinking of the RNA network can also be achieved by increasing the RNA content in the network. The use of a higher amount of RNA crosslinker for polymerization resulted in a greater compression modulus and a lower swelling ratio (Figure S9).

Chemical modifications of nucleic acids can increase the resistance of nucleic acids to enzymatic degradation by introducing structural alterations and steric hindrance.^42^ Inspired by this, we envisioned that the different degrees of acylation would result in distinct degradation behaviors of the RNA hydrogels. To test our hypothesis, we examined the biodegradability of the three RNA hydrogel variants (**100R**_**100**_, **50R**_**100**_, and **25R**_**100**_) under biologically relevant conditions (i.e., 15% fetal bovine serum, FBS) as shown in Figure 3. We noticed rapid and nearly complete degradation of **25R**_**100**_ and a relatively slow degradation of **50R**_**100**_, which was evidenced by the diffusion of GelRed to the supernatant. While **25R**_**100**_ and **50R**_**100**_ were completely degraded within 24 h, **100R**_**100**_ remained nearly intact for 18 days of incubation. The complete degradation of **100R**_**100**_ was finally observed after incubation for 30 days. These results suggest that the stability and durability of the RNA hydrogel can be engineered by selecting an appropriate RNA crosslinker, which could be useful for the controlled release of cargo loaded in the hydrogel.^43,44^

### Copolymerization of RNA Crosslinker with Acrylic Monomers

**Figure 4:**
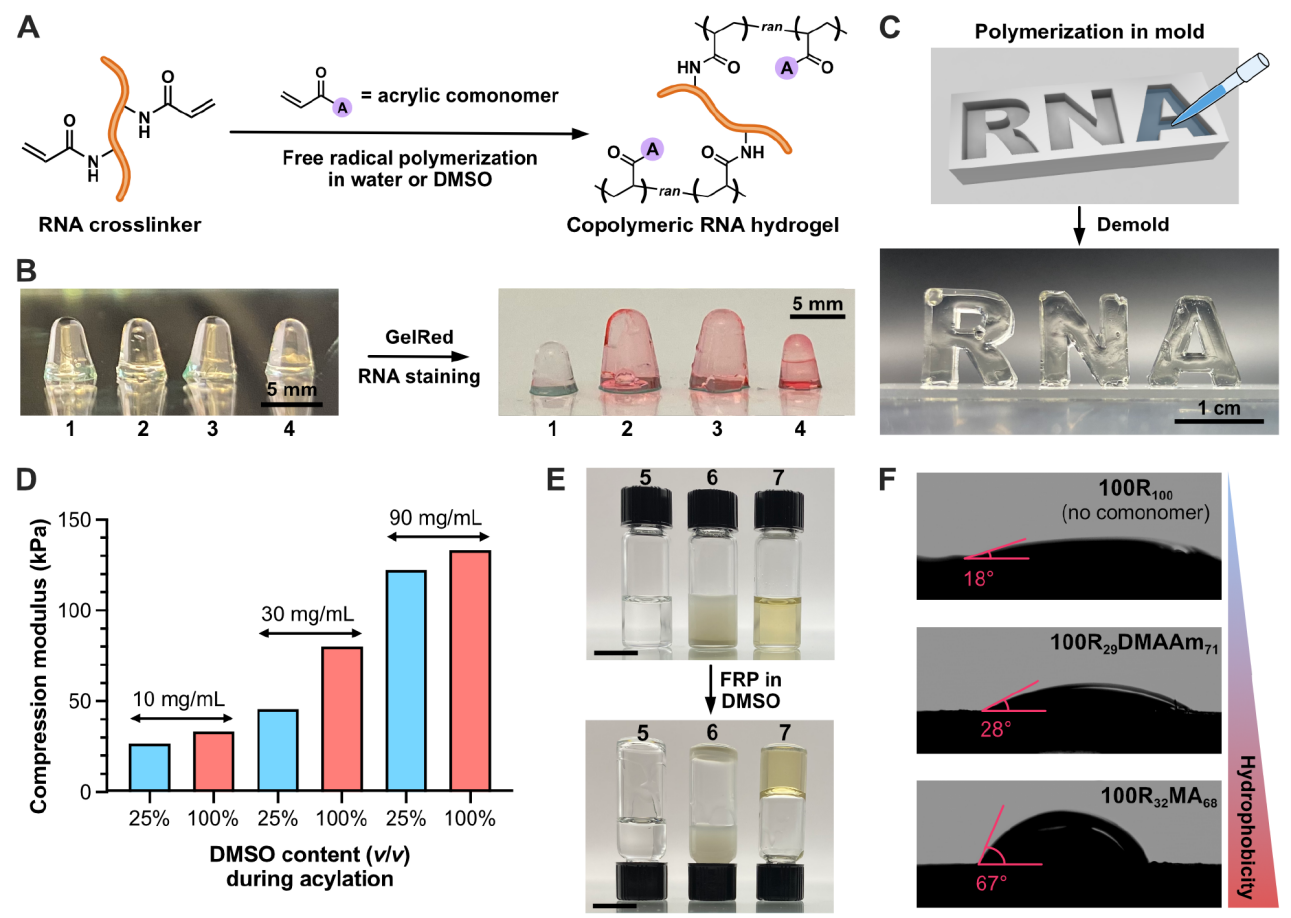
Copolymerization of RNA crosslinker in water and DMSO. (A) Scheme of copolymerization of RNA crosslinker. (B) Synthesis of RNA-NIPAM hybrid gels and staining with GelRed. (**1**–**4**) NIPAM gel without RNA (**1**), **25R**_**55**_**NIPAM**_**45**_ (**2**), **50R**_**55**_**NIPAM**_**45**_ (**3**), and **100R**_**55**_**NIPAM**_**45**_ (**4**), respectively. (C) RNA-acrylamide hybrid gel (**25R**_**67**_**AAmix**_**33**_) synthesized in the custom-designed mold. (D) Comparison of compression modulus of RNA-acrylamide hybrid gels with 10, 30, or 90 mg/mL of RNA crosslinkers synthesized under different acylation conditions (25% or 100%). Stress–strain curves for the calculation of compression moduli are shown in Figure S12. (E) Synthesis of the RNA-methyl acrylate (MA) hybrid gel in DMSO. MA with Irgacure 2959 (left), MA and unmodified RNA with Irgacure 2959 (middle), and MA and RNA X-linker with Irgacure 2959 (right, **100R**_**32**_**MA**_**68**_). Scale bars: 1 cm. (F) Contact angle test of RNA hydrogels with different surface polarities. Summaries of the reaction conditions for the hydrogel synthesis in water (Figure 4B–D) and in DMSO (Figure 4E,F) are shown in Tables S3 and S4, respectively.

To demonstrate the versatility of our method, we investigated the copolymerization of the RNA crosslinker with various acrylic monomers (Figure 4A). RNA crosslinkers synthesized under different concentrations of DMSO (25, 50, and 100% v/v) were copolymerized with *N*-isopropylacrylamide (NIPAM) to make RNA-NIPAM hybrid gels (**2**–**4** in Figure 4B). To facilitate abbreviated nomenclature, the RNA hybrid gels are referred to as ***x*R**_***y***_**Comonomer**_***z***_, where *x* is the % DMSO (v/v) used in the acylation process, “Comonomer” represents the abbreviation of the comonomer (e.g., NIPAM), and *y* and *z* indicate the weight percentages of RNA and the comonomer in the gel, respectively. After the successful copolymerization of RNA crosslinkers with NIPAM, we stained the hydrogels with GelRed (Figure 4B) by soaking the hydrogels in 20–100× GelRed in water. After overnight incubation under gentle shaking, all three RNA-NIPAM hybrid gels were stained by GelRed, which confirmed the presence of RNA in the hydrogels. Interestingly, water absorption and swelling of **25R**_**55**_**NIPAM**_**45**_ (**2** in Figure 4B) and **50R**_**55**_**NIPAM**_**45**_ (**3** in Figure 4B) was observed, thereby indicating a lower degree of crosslinking caused by the lower acrylamide content in the RNA crosslinkers synthesized in 25% and 50% DMSO (v/v). We also copolymerized NIPAM with an acrylamide/bis(acrylamide) 29:1 mix (AAmix) at a final concentration of 5% to make a NIPAM gel without RNA (**1** in Figure 4B). Despite the following staining with GelRed dye, the NIPAM gel remained unstained because of the absence of RNA. Similarly, under UV light (λ = 365 nm), strong fluorescence of RNA-acrylamide hybrid gels after treatment with RNA staining dyes was observed, which clearly indicated the RNA content in the hybrid gels (Figure S10). The degradation of the RNA-NIPAM copolymeric hydrogel in 15% FBS was also examined (Figure S11 and Table S2). The copolymeric hybrid RNA gels exhibited retarded degradation, possibly attributed to a reduced RNA content within the gel and steric hindrance.

We also carried out a reaction in a custom-designed mold by copolymerizing RNA crosslinker using the AAmix as the diluent (Figure 4C). The mold was charged with RNA crosslinker synthesized in 25% DMSO (v/v) and AAmix (final concentration of 2%) followed by the addition of APS and TEMED to initiate FRP. After 5 min of incubation, the RNA-acrylamide hybrid gel (**25R**_**67**_**AAmix**_**33**_) was demolded. The resulting copolymeric RNA hydrogels exhibited solid structural integrity, thereby enabling them to free-stand and maintain their shape in the air as a result of successful copolymerization with AAmix.

To quantify the RNA crosslinker-assisted enhancement of stiffness, we fabricated cylindrical RNA-acrylamide hybrid gels and conducted mechanical property tests in the compression mode (Figures 4D and S12). RNA crosslinkers synthesized in 25% or 100% DMSO (v/v) were copolymerized with 8% AAmix at the final concentrations of 10, 30, and 90 mg/mL, respectively. Figure 4D demonstrates that increasing the amount of RNA crosslinker in RNA-AAmix hybrids led to higher compression moduli of the copolymeric hydrogels, thereby indicating that the addition of RNA crosslinker reinforced the mechanical properties of the hybrid gels. The use of RNA crosslinker synthesized in a higher concentration of DMSO facilitated the formation of more strongly crosslinked networks, which is consistent with previous findings. In contrast, the addition of unmodified biomass RNA (**R**_**11**_**AAmix**_**89**_) showed no significant improvement in the mechanical properties (Figure S12A).

In addition to NIPAM and acrylamides, the capability to copolymerize hydrophobic monomers with the RNA crosslinker offers the potential to engineer a wide range of material properties, including enhanced stability in organic solvents and hydrophobic characteristics. Importantly, we observed the high solubility of the RNA crosslinker in DMSO (>200 mg/mL), which prompted us to investigate the copolymerization of the RNA crosslinker with hydrophobic monomers in organic solvent (i.e., DMSO). As shown in Figure 4E, the RNA crosslinker was mixed with methyl acrylate (MA) and copolymerized in DMSO by photoinduced FRP (λ = 365 nm) using Irgacure 2959 as the initiator. After 30 min of polymerization under UV light, the RNA-MA hybrid material, **100R**_**32**_**MA**_**68**_ (**7** in Figure 4E), was successfully fabricated. In contrast, **5** (MA with Irgacure 2959) or **6** (MA and unmodified RNA with Irgacure 2959) in Figure 4E did not form a gel, thereby indicating the successful formation of a polymeric network assisted by the RNA crosslinker. In addition to MA, a variety of other acrylic monomers with distinct hydrophobicity were also successfully copolymerized with the RNA crosslinker and characterized by Raman spectroscopy (Figure S13) and thermogravimetry (Figure S14) to confirm the identity of the copolymeric material. As shown in Figure 4F, we further conducted the contact angle measurements of the RNA hydrogel (**100R**_**100**_) and copolymeric RNA hybrid materials made with dimethyl acrylamide (**100R**_**29**_**DMAAm**_**71**_) or MA (**100R**_**32**_**MA**_**68**_). The distinct contact angles of the hydrogels imply that the copolymerization of appropriate acrylic monomers with the RNA crosslinker could tune the hydrophobicity of the RNA hydrogels.

### Polymerization of Methacrylic RNA Crosslinker through Reversible Deactivation Radical Polymerization

Reversible deactivation radical polymerization (RDRP) methods are changing the world by enabling the precise synthesis of polymers and materials in a property-controlled manner.^45^ Atom transfer radical polymerization (ATRP) and reversible addition–fragmentation chain transfer (RAFT) polymerization are among the most widely used RDRP techniques enabling precise control over the properties of synthetic polymers, including molecular weights, molecular weight distributions, composition, and architectures (e.g., multiblock copolymers, star-shaped polymers, etc.).^46−52^ As a result, polymeric networks and multiscale materials made by ATRP and RAFT have found various applications in electronics, adhesives, coatings, lubricants, and healthcare.^50^

This inspired us to investigate the possibility of polymerizing the RNA crosslinker through ATRP and RAFT, which could provide an alternative route to engineer the material properties, in addition to using different comonomers. To ensure well-controlled polymerization via ATRP and RAFT,^51,53^ we designed another acylating reagent with methacrylate functionalities using hydroxyethyl methacrylate (HEMA) and CDI (Figure S15). The resulting HEMA-functionalized imidazole carbamate (**HEMA-CM**) was treated to biomass RNA in 50% DMSO in water (v/v) overnight. Characterization of the methacrylic RNA crosslinker product using UV–vis (Figure S16) and ^1^H NMR spectroscopy (Figure S17) showed slightly reduced functionalization efficiency of **HEMA-CM** (3.6 methacrylate groups per 10 ribonucleotides) compared with **AAm-AI** (5.5 acrylamide groups per 10 ribonucleotides, Figure 2B).

**Figure 5:**
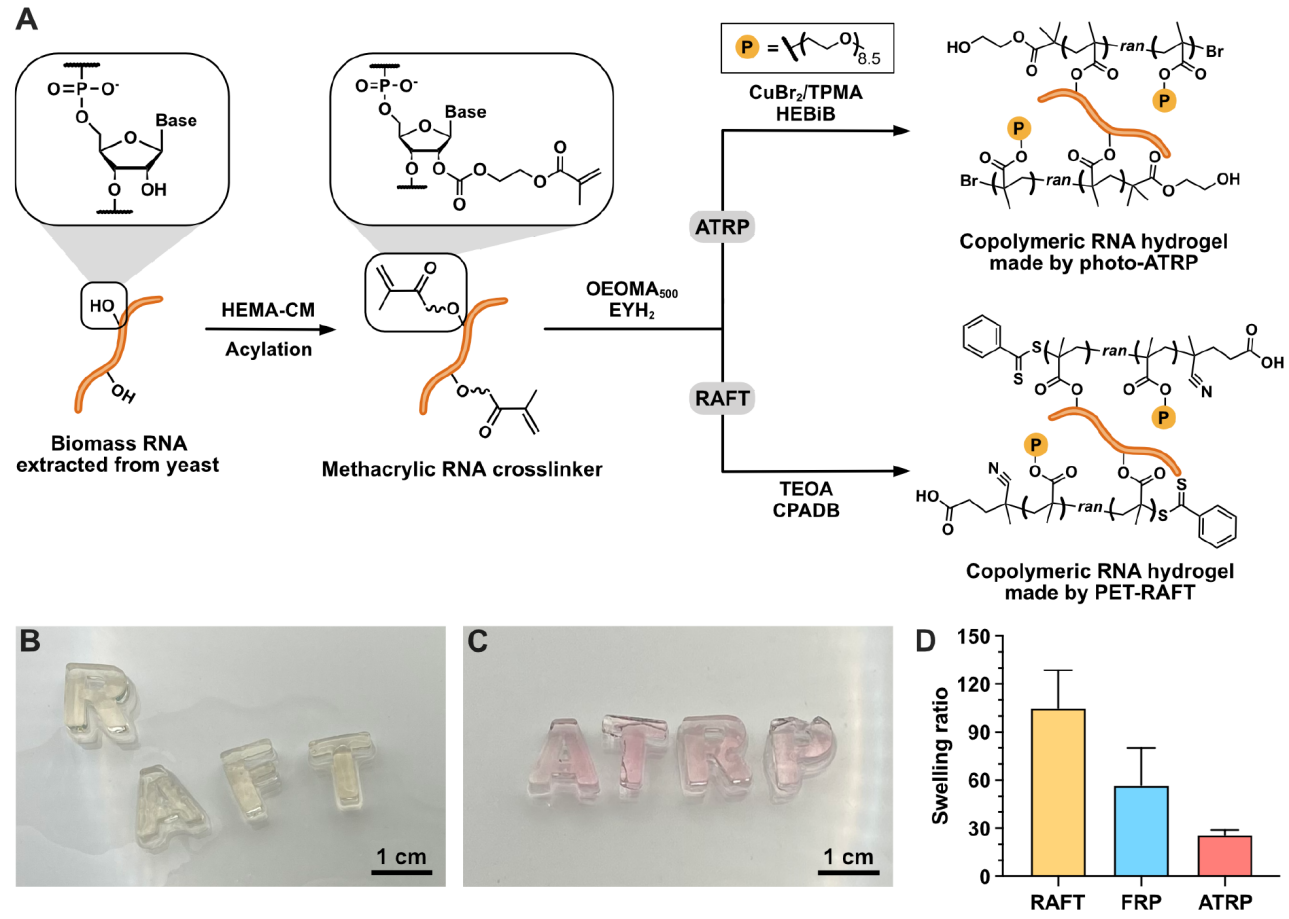
Methacrylic RNA crosslinker and copolymerization by different radical polymerization methods. (A) Scheme of reaction of biomass RNA with **HEMA-CM** reagent and subsequent copolymerization with OEOMA_500_ via EY/Cu-mediated photo-ATRP or PET-RAFT under green light irradiation (λ = 540 nm). (B–C) Digital camera image of RNA-OEOMA_500_ hybrid hydrogels fabricated by (B) PET-RAFT; and (C) photo-ATRP, respectively. (D) The swelling ratio of RNA-OEOMA_500_ hybrid hydrogels made by PET-RAFT, FRP, and photo-ATRP. The standard deviation was calculated from 3 different batches. A summary of the reaction conditions for the synthesis of the hydrogels is shown in Table S5. Abbreviations: TPMA [tris(2-pyridylmethyl)amine]; HEBiB (2-hydroxyethyl 2-bromoisobutyrate); TEOA (triethanolamine); CPADB [4-cyano-4-(phenylcarbonothioylthio)pentanoic acid].

The methacrylic RNA crosslinker was then polymerized by photo-ATRP^54−56^ or photoinduced electron/energy transfer RAFT polymerization (PET-RAFT)^57−59^ in PBS under green light irradiation using eosin Y (EYH_2_) and oligo(ethylene oxide) methyl ether methacrylate (OEOMA_500_, *M*_n_ = 500) as the photocatalyst and the model comonomer, respectively (Figure 5A). A notable photobleaching of eosin Y was observed after 30 min of PET-RAFT polymerization (Figure 5B), which was consistent with previous findings.^54^ In contrast, the RNA hydrogel made by EY/Cu-mediated photo-ATRP retained its pink color originating from the EYH_2_ dye (Figure 5C). This is due to the rapid electron transfer from excited eosin Y in the triplet state to the [Cu^II^/L]^2+^ complex.^54^ Next, the swelling ratio of the three hydrogels made by PET-RAFT, photo-ATRP, and FRP was measured to compare the effects of the polymerization method on the network (Figure 5D). A significant difference in the swelling behavior was observed among the RNA-OEOMA_500_ hybrid networks prepared by PET-RAFT, photo-ATRP, and FRP. The distinct swelling ratios are likely due to the use of multihandle crosslinkers and the difference in the initiation efficiencies of each photopolymerization method.^60−62^ The rapid initiation of the photo-ATRP process, facilitated by the efficient electron transfer to the [Cu^II^/L]^2+^ complex, led to a simultaneous and accelerated polymerization process, which led to a more efficient crosslinking, smaller network mesh size, and lower swelling ratio.

### Enhanced Electrical Conductivity Induced by Silver Doping

**Figure 6:**
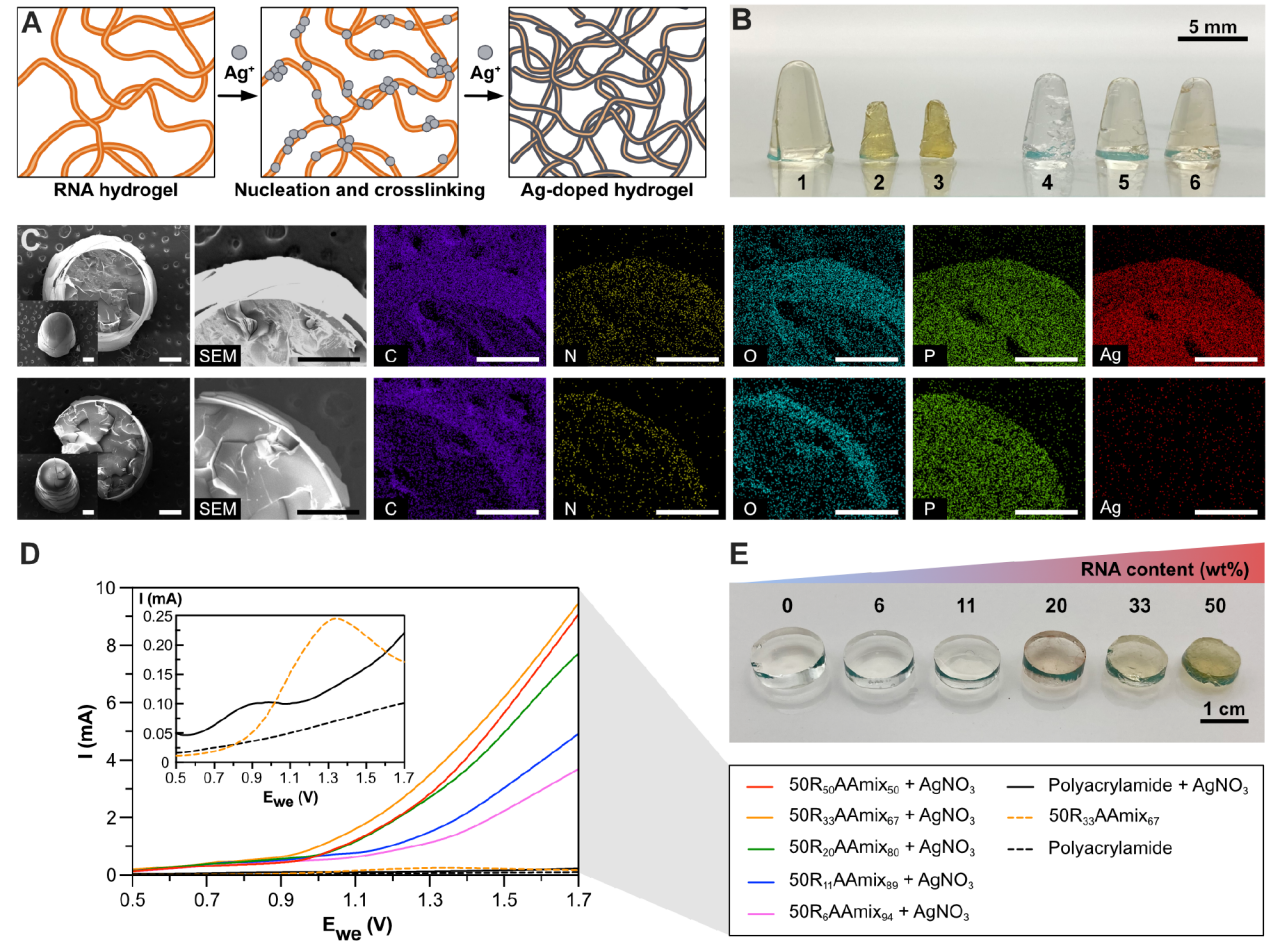
Silver-doping as the route to grant electrical conductivity to the RNA hydrogel. A summary of the reaction conditions for the synthesis of the hydrogels is shown in Table S6. (A) Schematic illustration of the binding, nucleation, and RNA-templated growth of Ag in the RNA hydrogel. (B) Digital camera image of AgNO_3_-treated polyacrylamide gels with or without RNA. (**1**–**3**) **50R**_**50**_**AAmix**_**50**_ after treatment with 0 (**1**), 100 (**2**), and 400 mM (**3**) AgNO_3_ for 3 h, respectively. (**4**–**6**) Polyacrylamide gels after treatment with 0 (**4**), 100 (**5**), and 400 mM (**6**) AgNO_3_ for 3 h, respectively. (C) SEM and EDX element mapping of RNA-acrylamide hydrogel. Top lane: RNA-acrylamide hydrogel (**2** in Figure 6B). Bottom lane: polyacrylamide hydrogel (**5** in Figure 6B). The inset in the SEM image at the leftmost is the SEM image of the hydrogel before cross-sectional cutting. Scale bars = 500 μm. (D) Electrical conductivity measurement of RNA-acrylamide hydrogels with different RNA content (0–50 wt %) after overnight treatment with 100 mM AgNO_3_. (E) Digital camera image of the AgNO_3_-treated RNA hydrogels used for the electrical conductivity test. From left to right, polyacrylamide gel, **50R**_**6**_**AAmix**_**94**_, **50R**_**11**_**AAmix**_**89**_, **50R**_**20**_**AAmix**_**80**_, **50R**_**33**_**AAmix**_**67**_, and **50R**_**50**_**AAmix**_**50**_, respectively.

Nucleic acids interact with transition metal ions, such as Au, Ag, Pt, and Co, through diverse mechanisms, including coordination, intercalation, and electrostatic interaction.^63^ The resulting metalized nucleic acids possess several applications, such as electrochemiluminescence probes,^64^ catalysis,^65,66^ and nanopatterning.^67−69^ We hypothesized that the metallization of RNA (e.g., coordination of metal ions to nucleobases and the subsequent reduction of metal by electron-rich nucleobases) could also occur on the polymeric RNA network (Figure 6A), as previously observed in the DNA-based materials.^20,70,71^ To test our hypothesis, we synthesized RNA-acrylamide copolymeric gels (**50R**_**50**_**AAmix**_**50**_) by FRP and washed the gel by soaking it in water overnight under gentle shaking. The gel was then treated with 400 mM AgNO_3_ (**3** in Figure 6B), 100 mM AgNO_3_ (**2** in Figure 6B), or water (**1** in Figure 6B), respectively. Interestingly, the shrinking of **50R**_**50**_**AAmix**_**50**_ was noticed after the 3 h of AgNO_3_ treatments, which may be attributed to the crosslinking induced by the formation of Cytosine–Ag^+^–Cytosine bridges.^72,73^ Additionally, a decrease in electrostatic repulsion between RNA strands resulting from the binding and reduction of metal ions around the RNA backbone also contributed to the contraction of the nucleic acid-based network, as observed in Au-doped DNA hydrogel.^20^ In contrast, the treatment of AgNO_3_ with the polyacrylamide gels did not result in noticeable shrinking of the gel (**4**–**6** in Figure 6B), which suggests the RNA-selective coordination of Ag^+^. As shown in Figure 6C, we further characterized the AgNO_3_-treated RNA gel (**2** in Figure 6B) and the AgNO3-treated polyacrylamide gel (**5** in Figure 6B) using scanning electron microscope (SEM). The cross-sectional energy dispersive X-ray (EDX) analysis of **2** (Figure 6C, top lane) and **5** (Figure 6C, bottom lane) confirmed the RNA-selective binding of Ag^+^. The elemental analysis presented in Figure S18 shows that treatment with a higher concentration of AgNO_3_ may result in a different loading of Ag in the hydrogel.

By harnessing the metal doping onto the RNA hydrogel, we envision various applications ranging from diagnostics,^74^ catalysis,^65,66^ and electronic devices.^18^ As an example, we conducted measurements to assess the conductivity of the RNA-acrylamide hybrid gels (Figure 6D,E). We used different RNA contents (0–50 wt %) to synthesize these gels and incubated them in 100 mM AgNO_3_ overnight. The successful binding of Ag^+^ to RNA and crosslinking was evidenced by the shrinking of the RNA hydrogels (Figure 6E). We placed the dried hydrogels between two stainless steel plates in CR2032-type coin cells for conductivity comparison. As demonstrated in the current–voltage (*I*–*V*) measurement results in Figure 6D, the AgNO_3_ treatment played a crucial role in inducing the conductivity of the RNA hydrogel. Moreover, we observed stronger conductivity when more RNA was used in hydrogel fabrication. These results suggest that the treatment of metal precursor solutions may provide an additional possibility to engineer the hydrogel property in a postsynthetic manner.

## Conclusions

In summary, we report the successful conversion of biomass RNA into an acrylic crosslinker using acyl imidazole chemistry. The number of acrylic moieties on the RNA was engineered by varying the acylation conditions (% DMSO v/v in water and the amount of **AAm-AI**), which allows for the control over the mechanical properties and degradability of the network. Increasing the RNA crosslinker concentration can also serve as an alternative method for achieving a densely crosslinked network, which results in enhanced stiffness and a lower swelling ratio. The resulting acrylamido RNA crosslinker could undergo radical polymerization with a diverse range of other acrylic monomers to regulate the surface polarity of the copolymeric RNA hydrogel while also allowing the control over the RNA content (0–100 wt %) within the gel. Our study also explored the polymerization of the methacrylic RNA crosslinker via RDRP techniques (i.e., photo-ATRP and PET-RAFT), which enables the fabrication of copolymeric RNA gels with customizable swelling ratios through the choice of the polymerization method. The metallization of RNA with silver ions demonstrated its potential to enhance the electrical conductivity of biomass RNA-based materials. This work expands the possibilities for biomass RNA-based material fabrication with tailored properties while overcoming the challenges associated with conventional synthetic strategies. This technique is expected to accelerate advancements in the emerging field of biomass nucleic acid-based materials, thereby enabling diverse applications, such as drug release, electronics, and catalysis.

## Methods

### General Procedure for the Synthesis of **AAm-AI**

In 650 μL of DMSO was dissolved 161.2 mg (1 mmol) of CDI. To the dissolved CDI in DMSO, 185.2 mg (1 mmol) of 6-acrylamidohexanoic acid was added, and the final volume was brought to 1 mL by the addition of DMSO. The resulting **AAm-AI** stock (1 M) was incubated at room temperature for 30 min under gentle shaking.

### General Procedure for the Synthesis of the Acrylamido RNA Crosslinkers

With 400 μL of 1 M **AAm-AI** stock was mixed a 40 mg portion of yeast RNA. For the acylation under the 25% or 50% v/v DMSO in water, an additional 1200 or 400 μL of nuclease-free water was added, respectively. After the overnight incubation at room temperature under gentle shaking, the resulting RNA crosslinker was precipitated by the addition of 3 M sodium acetate (1/10 volume) and isopropanol (1.5 volume). The precipitated RNA crosslinker was isolated by centrifugation (13 000 rpm, 15 min) at 4 °C. The isolated RNA crosslinker pellet was redissolved in water and further purified by additional precipitation and centrifugation. Finally, the purified RNA crosslinker pellet was dissolved in water, and the concentration of RNA (mg/mL) was determined by measuring A_260_ [extinction coefficient = 40 (μg/mL)^−1^ cm^–1^]. To estimate of the degree of acylation, ∼3 mg of RNA crosslinker was dissolved in 600 μL of D_2_O followed by ^1^H NMR analysis.

### General Procedure for the Fabrication of RNA Hydrogels via FRP in Water

To homopolymerize the RNA crosslinker in a reaction volume of 50 μL, acrylamido RNA crosslinker (final concentration of 150–250 mg/mL) was taken, and the volume was adjusted to 44 μL by adding water. Subsequently, 1 μL of TEMED and 5 μL of 10% APS in water were added, and the mixture was thoroughly mixed. The resulting mixture was incubated at room temperature for 3 min to polymerize. For the copolymerization of the RNA crosslinker, 3 M NIPAM stock (339.5 mg in 1 mL of DMSO) or 40% acrylamide mix [acrylamide/bis(acrylamide) = 29:1] was prepared. Next, acrylamido RNA crosslinker (final concentration of 5–90 mg/mL) was mixed with NIPAM stock (final concentration of 250 mM) or acrylamide mix (final concentration of 2–8%), and the volume was brought to 44 μL by adding water. Subsequently, 1 μL of TEMED and 5 μL of 10% APS in water were added and thoroughly mixed. The resulting mixture was incubated at room temperature for 3 min to polymerize.

## Supporting Information Available

Additional experimental details and information include NMR spectra, SEC-MALS traces, compressive stress–strain curves, Raman spectra, TGA plots, UV–vis spectra, digital camera images, EDX spectra, and tables for the reaction conditions and results summary (PDF)
