# Supplementary material for: Biomass RNA for the Controlled Synthesis of Degradable Networks by Radical Polymerization
Source: ACS Nano. 2023 Oct 18;17(21):21912–22. doi: 10.1021/acsnano.3c08244 (PMC10655241; doi:10.1021/acsnano.3c08244)
Supplement: Supplementary file 1 — nn3c08244_si_001.pdf [file nn3c08244_si_001.pdf]

Supporting information

Biomass RNA for the Controlled Synthesis of Degradable Networks by Radical Polymerization

Jaepil Jeong,[1,2] So Young An,[1] Xiaolei Hu,[1] Yuqi Zhao,[3] Rongguan Yin,[1] Grzegorz Szczepaniak,[1,4] Hironobu Murata,[1] Subha R. Das,[1,2]* and Krzysztof Matyjaszewski,[1]*

[1]Department of Chemistry, Carnegie Mellon University, Pittsburgh, PA 15213, United States.

[2]Center for Nucleic Acids Science & Technology, Carnegie Mellon University, Pittsburgh, PA 15213, United States

[3]Department of Materials Science & Engineering, Carnegie Mellon University, Pittsburgh, PA 15213, United States

[4]University of Warsaw, Faculty of Chemistry, Pasteura 1, 02-093 Warsaw, Poland

*Correspondence: S.R.D (srdas@andrew.cmu.edu); K.M. (km3b@andrew.cmu.edu)

Table of Contents

Experimental details S3
Materials S3
Instruments S3
Procedures S3
General procedure for the synthesis of AAm-AI S3
General procedure for the acrylamido RNA crosslinker synthesis S4
General procedure for the characterization of RNA crosslinker using NMR S4
General procedure for the fabrication of RNA hydrogel via free radical polymerization in water S4
Degradation in FBS S5
RNA crosslinker-assisted engineering of mechanical property S5
General procedure for the FRP of RNA crosslinker with comonomer in DMSO S5
General procedure for the synthesis of HEMA-CM S6
General procedure for the methacrylic RNA crosslinker synthesis S6
General procedure for the RAFT and ATRP of RNA crosslinker with $OEOMA_{500}$ in mold S6
Determination of the swelling ratio of RNA-OEOMA500 hybrid gels made by RAFT, ATRP, and FRP S7
RNA-acrylamide hybrid gels doped with $AgNO_3$ for SEM-EDX analysis S8
RNA-acrylamide hybrid gels doped with $AgNO_3$ for electrical conductivity test S8
Supplementary Data S10
Supplementary Tables S28

Experimental details

Materials

All chemicals, including torula yeast RNA (type VI), were purchased from Sigma Aldrich unless stated otherwise. 6-Acrylamidohexanoic acid and carbonyldiimidazole (CDI) was purchased from TCI America. 40% acrylamide:bisacrylamide mix (29:1), tetramethylethylenediamine (TEMED), and 10X phosphate-buffered saline (PBS) were purchased from Fisher Scientific. 96 well plate was purchased from Greiner (CellStar ®). Fetal bovine serum (FBS) was purchased from Gibco. GelRed was purchased from Biotium. Chemical-resistant slippery sheet made from Teflon® PTFE were purchased from McMaster-Carr, carved, and used as the mold for polymerization.

Instruments

NMR experiments were conducted using a Bruker Avance NEO NMR spectrometer, equipped with a cryogenically cooled Prodigy CPP BBO BB-H&F-D probehead, operating at 500 MHz for ^{1}H and 125.73 MHz for ^{13}C. The UV-Vis absorbance spectra were obtained using a NanoDrop One UV-Vis spectrophotometer (ThermoFisher Scientific). The mass spectra of biomass RNA were obtained using the UltrafleXtreme MALDI-TOF Mass Spectrometer from Bruker, with the MTP 384 Target Plate Ground Steel. RSA-G2 Solids Analyzer (TA Instruments) was used for the mechanical property analysis in compression mode. The fabrication of biomass RNA gel via PET-RAFT or EY-ATRP under green light was carried out in a 96-well plate using Lumidox II 96-Well LED Arrays ($\lambda = 540$ nm) and controlled by the Lumidox II Controller. MacroRAM™ Raman spectrometer (Horiba Scientific) equipped with a 0.25-inch diameter fiber ball probe (Marqmetrix) was used to obtain Raman spectra under the laser excitation at 785 nm. VCA Optima (AST Products Inc.) was used for contact angle measurement and the result was analyzed using VCA Optima XE software. Electrochemical measurements were performed using a Bio-Logic 16-channel VMP-3 multi-channel potentiostat/electrochemical spectrometer at room temperature. Thermogravimetric analysis (TGA) was performed on a TGA 550 (TA Instrument) and the data was processed by using TA Universal Analysis software. Scanning electron microscope (SEM) was performed on a Quanta 600 FEG instrument with XMAX 80mm SDD EDX detector.

Procedures

General procedure for the synthesis of AAm-AI

161.2 mg (1 mmol) of CDI was dissolved in 650 µL of DMSO. To the dissolved CDI in DMSO, 185.2 mg (1 mmol) of 6-acrylamidohexanoic acid was added and the final volume was brought to 1 mL by the addition of DMSO. The resulting AAm-AI stock (1 M) was incubated at room temperature for 30 min under gentle shaking.

General procedure for the synthesis of acrylamido RNA crosslinkers

40 mg of yeast RNA was mixed with 400 µL of 1M **AAm-AI** stock. For the acylation under the 25% or 50% *v/v* DMSO in water, an additional 1200 µL or 400 µL of nuclease-free water was added, respectively. After the overnight incubation at room temperature under gentle shaking, the resulting RNA crosslinker was precipitated by the addition of 3M sodium acetate (1/10 volume) and isopropanol (1.5 volume). The precipitated RNA crosslinker was isolated by centrifugation (13000 rpm, 15 min) at 4 °C. The isolated RNA crosslinker pellet was redissolved in water and further purified by additional precipitation and centrifugation. Finally, the purified RNA crosslinker pellet was dissolved in water and the concentration of RNA (mg/mL) was determined by measuring A_{260} (extinction coefficient = 40 $(\mu g/mL)^{-1}cm^{-1}$). To estimate of the degree of acylation*, ca.* 3 mg of RNA crosslinker was dissolved in 600 µL of D_2O followed by ^{1}H NMR analysis.

General procedure for the characterization of RNA crosslinker using NMR

The ^{1}H, ^{13}C-HSQC experiment was performed using the hsqcedetgpsp.3 pulse program from the Bruker library, with the following parameters: TD (2048 in F2, 128 in F1), SW[ppm] (10 F2, 180 in F1), O1P[ppm] (5 in F2, 90 in F1), AQ[s] (0.2048 in F2, 0.00282 in F1), NS=16, D1=1 s. Processed data had SI of 2048 in F2 and 2048 in F1).
The ^{1}H-DOSY experiment utilized the ledbpgp2s pulse program from the Bruker library, with these parameters: TD (25000 in F2, 16 in F1), SW(F2)=10 ppm, O1P=5 ppm, AQ=2.5 s, D1=1 s, p30 (Little Delta)=5 ms, D20 (Big Delta)=200 ms. The probe's maximum gradient strength is 65.7 Gauss/cm, with an effective strength of 59.1 Gauss/cm (100%) when considering the use of Smoothed Square (SMSQ10.100) gradient pulses (integral factor of 0.9). A linear gradient ramp of 16 points (TD in F1) ranging from 2% to 98% (1.2 to 57.9 Gauss/cm) of the maximum gradient strength was employed.

General procedure for the fabrication of RNA hydrogels via free radical polymerization in water

To homopolymerize the RNA crosslinker in a reaction volume of 50 µL, acrylamido RNA crosslinker (final concentration of 150–250 mg/mL) was taken and the volume was adjusted to 44 µL by adding water. Subsequently, 1 µL of TEMED and 5 µL of 10% APS in water were added and thoroughly mixed. The resulting mixture was incubated at room temperature for 3 min to polymerize. For the copolymerization of the RNA crosslinker, 3 M NIPAM stock (339.5 mg in 1 mL DMSO) or 40% acrylamide mix (acrylamide:bisacrylamide = 29:1) was prepared. Next, acrylamido RNA crosslinker (final concentration of 5–90 mg/mL) was mixed with NIPAM stock (final concentration of 250 mM) or acrylamide mix (final concentration of 2–8%) and the volume was brought to 44 µL by adding water. Subsequently, 1 µL of TEMED and 5 µL of 10% APS in water were added and thoroughly mixed. The resulting mixture was incubated at room temperature for 3 min to polymerize.

Degradation in FBS

Acrylamido RNA crosslinker was synthesized in 100%, 50%, or 25% *v/v* DMSO in water following the procedure described above. Next, each RNA crosslinker was polymerized with or without addition of NIPAM (final concentration of 250 mM or 45 wt% of NIPAM) to synthesize homopolymerized RNA hydrogel (**100R$_{100}$**, **50R$_{100}$**, and **25R$_{100}$**) or RNA-NIPAM hybrid gel (**100R$_{55}$NIPAM$_{45}$**, **50R$_{55}$NIPAM$_{45}$**, and **25R$_{55}$NIPAM$_{45}$**) in a reaction volume of 25 µL. Next, the hydrogels were stained by incubation in 1.5 mL of 25–100X GelRed in water overnight. After the staining process, the hydrogels were washed by exchanging supernatant with fresh water 3 times or overnight incubation in fresh water. Finally, the hydrogels were soaked in 15% FBS in aqueous buffer (50 mM Tris-HCl, 75 mM KCl, 3 mM MgCl2, 10 mM DTT) and incubated at 37 °C.

RNA crosslinker-assisted engineering of mechanical property

Acrylamido RNA crosslinker was synthesized in 100% or 25% *v/v* DMSO in water following the procedure described above. Next, each RNA crosslinker was mixed (at the final concentration of 10, 30, or 90 mg/mL) with 9 µL of 40% acrylamide mix, and the volume was brought to 39.6 µL by adding water. Subsequently, 0.9 µL of TEMED and 4.5 µL of 10% APS in water were added and thoroughly mixed. The resulting mixture was incubated at room temperature for 3 min to polymerize.

For the mechanical property analysis, the cylindrical RNA-acrylamide hybrid gels with diameters of 5 mm and heights of *c.a.* 2.3 mm (i.e., volume of 45 µL) are tested by using TA RSA-G2 in the compression mode. The samples were compressed at a constant linear rate of 0.01 s^{-1} at room temperature. Compression moduli were calculated from the incipient slope of strain stress curves at 10% strain.

General procedure for the FRP of RNA crosslinker with comonomer in DMSO

Prior to polymerization, stock solutions of each comonomer and radical initiator were prepared as follows at the final volume of 1 mL.

3 M Methyl acrylate (MA)	:	258.3 mg of MA in DMSO
3 M Dimethylacrylamide (DMAAm)	:	297.4 mg of DMAAm in DMSO
3 M Hydroxyethyl acrylate (HEMA)	:	390.4 mg of HEMA in DMSO
3 M Butyl acrylate (BA)	:	384.5 mg of BA in DMSO
3 M Acrylonitrile (AN)	:	159.2 mg of AN in DMSO
0.5 M Irgacure 2959	:	112.1 mg of Irgacure 2959 in MeOH

Next, the acrylamido RNA crosslinker synthesized under the 100% DMSO condition was mixed (at the final concentration of 60 mg/mL) with 400 µL of desired monomer stock and the volume was adjusted to 784 µL by adding DMSO. Finally, 16 µL of 0.5 M Irgacure 2959 was introduced followed by irradiation of UV light for 30 min. For the characterization of the RNA hybrid hydrogels synthesized in DMSO, the hydrogels were soaked in excess THF or

EtOH for 24 h followed by drying in a desiccator overnight. Finally, the hydrogels were analyzed by Raman spectroscopy and contact angle meter.

General procedure for the synthesis of HEMA-CM

Prior to use, HEMA was passed through a short column of basic alumina to remove the inhibitor. Then, 260.3 mg (2 mmol) of the purified HEMA was mixed with 350 µL of DMSO. To the HEMA in DMSO, 324.3 mg of CDI (2 mmol) was introduced, and thoroughly mixed, and the volume was brought to 1 mL by adding DMSO. Finally, the mixture was incubated at room temperature for 30 min under gentle shaking and the resulting HEMA-functionalized imidazole carbamate (**HEMA-CM**) was analyzed by NMR spectroscopy.

General procedure for the methacrylic RNA crosslinker synthesis

40 mg of yeast RNA was mixed with 400 µL of water and 400 µL of 2M **HEMA-CM** stock (i.e., 50% *v*/*v* DMSO in water). After the overnight incubation at room temperature under gentle shaking, the resulting RNA crosslinker was precipitated by the addition of 3M sodium acetate (80 µL) and isopropanol (1200 µL) followed by centrifugation (13000 rpm, 15 min) at 4 °C. The isolated RNA crosslinker pellet was redissolved in water and further purified by additional precipitation and centrifugation. Finally, the purified RNA crosslinker pellet was dissolved in water and the concentration of RNA (mg/mL) was determined by measuring A_{260} (extinction coefficient = 40 $(\mu g/mL)^{-1}cm^{-1}$). For the estimation of the degree of acylation, *ca.* 3 mg of RNA crosslinker was mixed with 600 µL of D_2O followed by ^1H NMR analysis.

General procedure for the RAFT and ATRP of RNA crosslinker with $OEOMA_{500}$ in mold

For the polymerization of RNA crosslinker via PET-RAFT, stock solutions were prepared as follows:

100 mM CPADB stock	:	14.0 mg of CPADB in 0.5 mL of DMSO
100 mM TEOA stock	:	14.9 mg of TEOA in 1 mL of water
1.5 mM EYH_2 stock	:	4.86 mg of EYH_2 in 5 mL of DMSO
0.7 M $OEOMA_{500}$ stock	:	1750 mg of $OEOMA_{500}$ in 5 mL of water

Next, 571 µL of $OEOMA_{500}$ stock, 20 µL of CPADB stock, 12 µL of TEOA stock, 13.3 µL of EYH_2 stock, 100 µL of 10X PBS, 16.7 µL of DMSO and methacrylic RNA crosslinker synthesized in 50% *v*/*v* DMSO (3.6 acylation per 10 ribonucleotides) were mixed, and the final volume of the mixture was brought to 1 mL by adding water. The reaction mixture was placed in a mold followed by the irradiation of green light for 30 min.

Reaction condition: $[OEOMA_{500}]/[CPADB]/[TEOA]/[EYH_2]$ = 200/1/0.6/0.01, $[OEOMA_{500}]$ = 400 mM and [RNA crosslinker] = 75 mg/mL.

For the polymerization of RNA crosslinker via EY-ATRP, stock solutions were prepared as follows:

100 mM HEBiB stock	:	10.6 mg of HEBiB in 0.5 mL of water
56.2 mM $CuBr_2$ stock	:	25.1 mg of $CuBr_2$ in 2 mL of 50% *v*/*v* DMSO in water
336.3 mM TPMA stock	:	24.4 mg of TPMA in 0.25 mL of DMSO
1.5 mM EYH_2 stock	:	4.86 mg of EYH_2 in 5 mL of DMSO
0.7 M $OEOMA_{500}$ stock	:	1750 mg of $OEOMA_{500}$ in 5 mL of water

Next, 571 µL of $OEOMA_{500}$ stock, 20 µL of HEBiB stock, 7.11 µL of $CuBr_2$ stock, 3.57 µL of TPMA stock, 13.3 µL of EYH_2 stock, 100 µL of 10X PBS, 33 µL of DMSO and methacrylic RNA crosslinker synthesized in 50% *v*/*v* DMSO (3.6 acylation per 10 ribonucleotides) were mixed, and the final volume of the mixture was brought to 1 mL by adding water. The reaction mixture was placed in a mold followed by the irradiation of green light for 30 min.
Reaction condition: [$OEOMA_{500}$]/[HEBiB]/[$CuBr_2$]/[TPMA][EYH_2] = 200/1/0.2/0.6/0.01, [$OEOMA_{500}$] = 400 mM and [RNA crosslinker] = 75 mg/mL.

Determination of the swelling ratio of RNA-OEOMA500 hybrid gels made by RAFT, ATRP, and FRP

For the synthesis of RNA-$OEOMA_{500}$ hybrid gel via PET-RAFT, 68.6 µL of $OEOMA_{500}$ stock, 1.92 µL of CPADB stock, 1.44 µL of TEOA stock, 1.6 µL of EYH_2 stock, 12 µL of 10X PBS, 2.48 µL of DMSO and methacrylic RNA crosslinker synthesized in 50% *v*/*v* DMSO (3.6 acylation per 10 ribonucleotides) were mixed, and the final volume of the mixture was brought to 120 µL by adding water. The reaction mixture was placed in a 95-well plate followed by the irradiation of green light for 30 min using Lumidox II 96-Well LED Arrays (λ = 540 nm).
Reaction condition: [$OEOMA_{500}$]/[CPADB]/[TEOA]/[EYH_2] = 250/1/0.75/0.125, [$OEOMA_{500}$] = 400 mM and [RNA crosslinker] = 36.6 mg/mL.

For the synthesis of RNA-$OEOMA_{500}$ hybrid gel via EY-ATRP, 68.6 µL of $OEOMA_{500}$ stock, 1.92 µL of HEBiB stock, 1.92 µL of $CuBr_2$ stock, 0.96 µL of TPMA stock, 1.6 µL of EYH_2 stock, 12 µL of 10X PBS, 3.4 µL of DMSO and methacrylic RNA crosslinker synthesized in 50% *v*/*v* DMSO (3.6 acylation per 10 ribonucleotides) were mixed, and the final volume of the mixture was brought to 120 µL by adding water. The reaction mixture was placed in a 95-well plate followed by the irradiation of green light for 30 min using Lumidox II 96-Well LED Arrays (λ = 540 nm).
Reaction condition: [$OEOMA_{500}$]/[HEBiB/[$CuBr_2$]/[TEOA]/[EYH_2] = 250/1/0.56//1.68/0.125, [$OEOMA_{500}$] = 400 mM and [RNA crosslinker] = 36.6 mg/mL.

For the synthesis of RNA-$OEOMA_{500}$ hybrid gel via FRP, 68.6 µL of $OEOMA_{500}$ stock, 0.39 µL of Irgacure 2959 stock, 12 µL of 10X PBS, 3 µL of DMSO and methacrylic RNA crosslinker synthesized in 50% *v*/*v* DMSO (3.6 acylation per 10 ribonucleotides) were mixed, and the final volume of the mixture was brought to 120 µL by adding

water. The reaction mixture was placed in a 95-well plate followed by the irradiation of green light for 30 min using Lumidox II 96-Well LED Arrays (λ = 365 nm).
Reaction condition: [$OEOMA_{500}$]/[Irgacure 2959] = 250/1, [$OEOMA_{500}$] = 400 mM and [RNA crosslinker] = 36.6 mg/mL.

The freshly synthesized hydrogel was soaked in water for 18 h. Next, the swollen hydrogel was transferred to a glass vial and incubated at 60 °C for 18 h. The swelling ratio was calculated using the following equation and the standard deviation was calculated from 3 different batches:

$$Swelling\ ratio = \frac{Weight\ of\ swollen\ gel - weight\ of\ dried\ gel}{Weight\ of\ dried\ gel}$$

RNA-acrylamide hybrid gels doped with $AgNO_3$ for SEM-EDX analysis

Acrylamido RNA crosslinker was synthesized in 50% *v*/*v* DMSO in water following the procedure described above. Next, the RNA crosslinker was mixed (at the final concentration of 80 mg/mL) with 20 µL of 40% acrylamide mix (i.e., a final concentration of 8%) and the volume was brought to 88 µL by adding water. Subsequently, 2 µL of TEMED and 10 µL of 10% APS in water were added and thoroughly mixed. After the 3 min, the hydrogels were taken out from the reaction tube and soaked in water under gentle shaking overnight at room temperature. Next, the supernatant was removed and the gel was washed with fresh water 3 times, and immersed in 4 mL of 400 mM $AgNO_3$, 4 mL of 100 mM $AgNO_3$, or water, respectively. After 3 h of incubation at room temperature under gentle shaking, the gel was washed by exchanging the supernatant 3 times with water. The hydrogel was dried for 24 h in a desiccator at room temperature for the SEM-EDX analysis.

RNA-acrylamide hybrid gels doped with $AgNO_3$ for electrical conductivity test

Acrylamido RNA crosslinker was synthesized in 50% *v*/*v* DMSO in water following the procedure described above. Next, the RNA crosslinker was mixed (at the final concentration of 5–80 mg/mL) with 44 µL of 40% acrylamide mix (i.e., a final concentration of 8%) and the volume was brought to 193.6 µL by adding water. Subsequently, 4.4 µL of TEMED and 22 µL of 10% APS in water were added and thoroughly mixed. After the 3 min, the hydrogels were taken out from the reaction tube and soaked in water under gentle shaking overnight at room temperature. Next, the supernatant was removed and the hydrogel was washed with fresh water 3 times, and immersed in 10 mL of 100 mM $AgNO_3$. After overnight incubation at room temperature under gentle shaking, the gel was washed by exchanging the supernatant with water.
Electrochemical measurements were performed using a Bio-Logic 16-channel VMP-3 multi-channel potentiostat/electrochemical spectrometer at room temperature. Current-Voltage (IV) measurements were taken in a

voltage range of 0.4 - 2.0 V at 0.05 V/s scan rate by placing dried gels between two stainless steel (diameter *ca.* 20 mm) in CR2032-type coin cells.

Supplementary Data

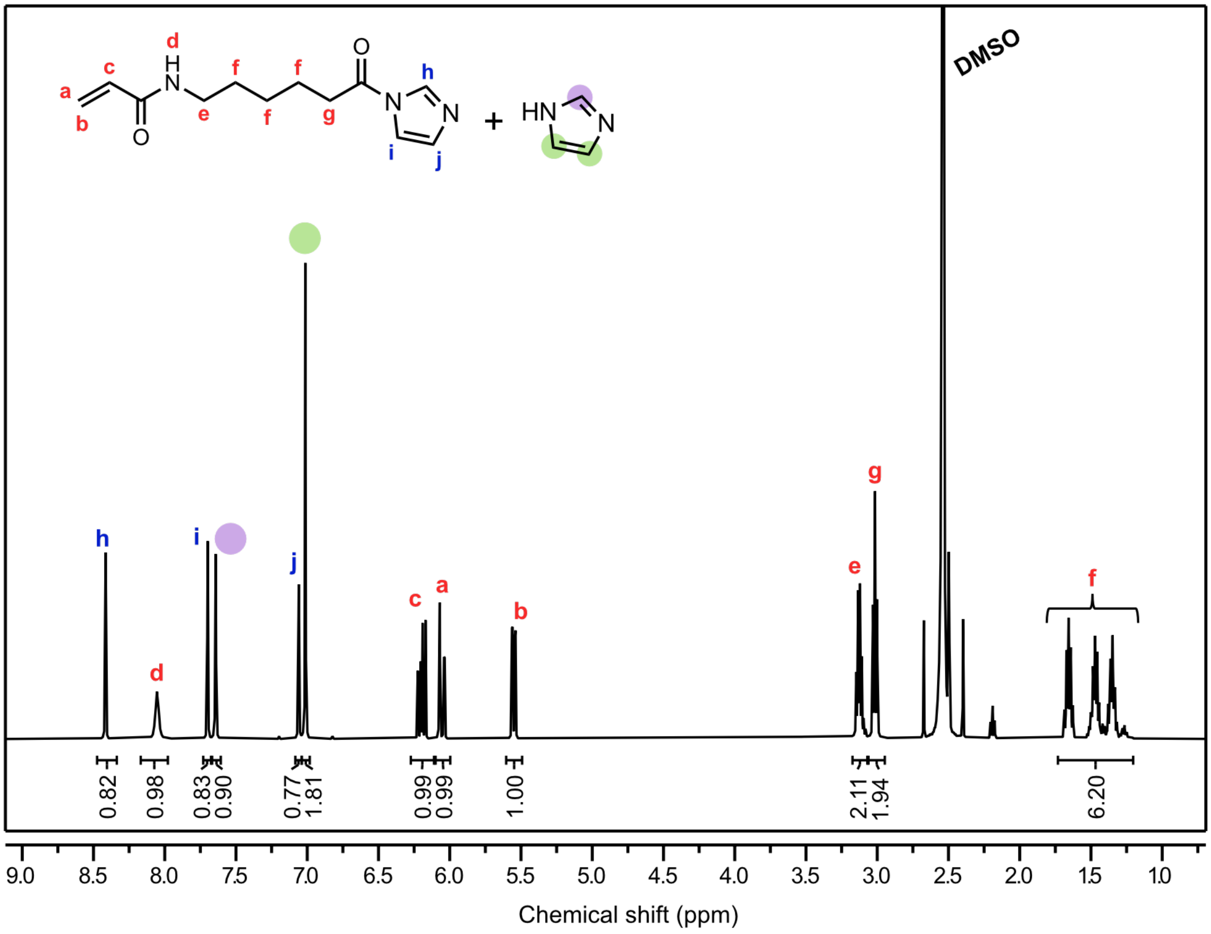

Figure S1. ^{1}H NMR spectrum **of AAm-AI**. ^{1}H NMR (500 MHz, DMSO-d_6) δ 8.42 (t, J = 1.1 Hz, 1H), 8.05 (t, J = 5.9 Hz, 1H), 7.70 (t, J = 1.4 Hz, 1H), 7.64 (t, J = 1.0 Hz, 1H), 7.06 (dd, J = 1.6, 0.8 Hz, 1H), 7.01 (d, J = 1.0 Hz, 2H), 6.20 (dd, J = 17.1, 10.2 Hz, 1H), 6.05 (dd, J = 17.1, 2.3 Hz, 1H), 5.55 (dd, J = 10.1, 2.2 Hz, 1H), 3.13 (td, J = 6.9, 5.6 Hz, 2H), 3.02 (t, J = 7.3 Hz, 2H), 1.66 (p, J = 7.4 Hz, 2H), 1.57 – 1.40 (m, 3H), 1.40 – 1.29 (m, 2H).

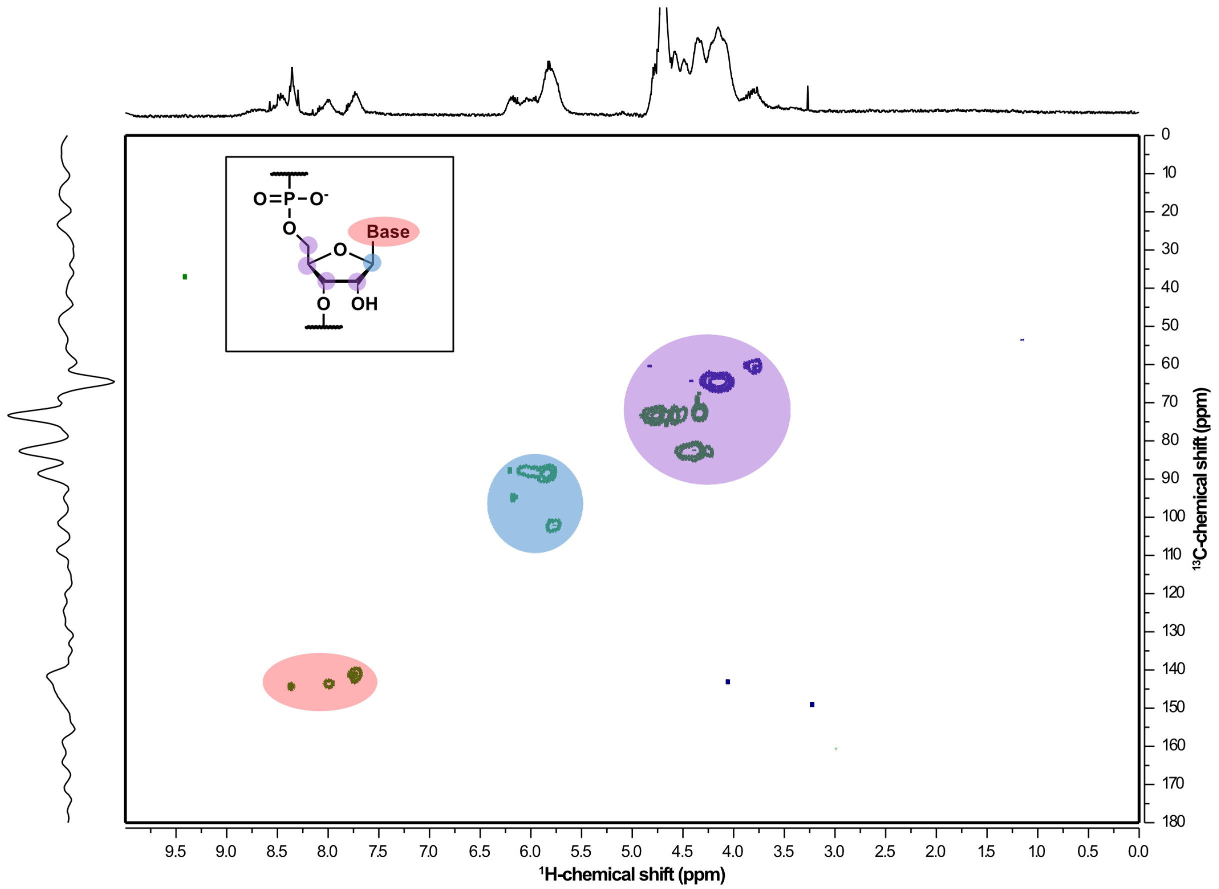

Figure S2. 2D ^{1}H–^{13}C HSQC spectra of unmodified biomass RNA. Green peaks represent CH and blue peaks represent CH_2, respectively.

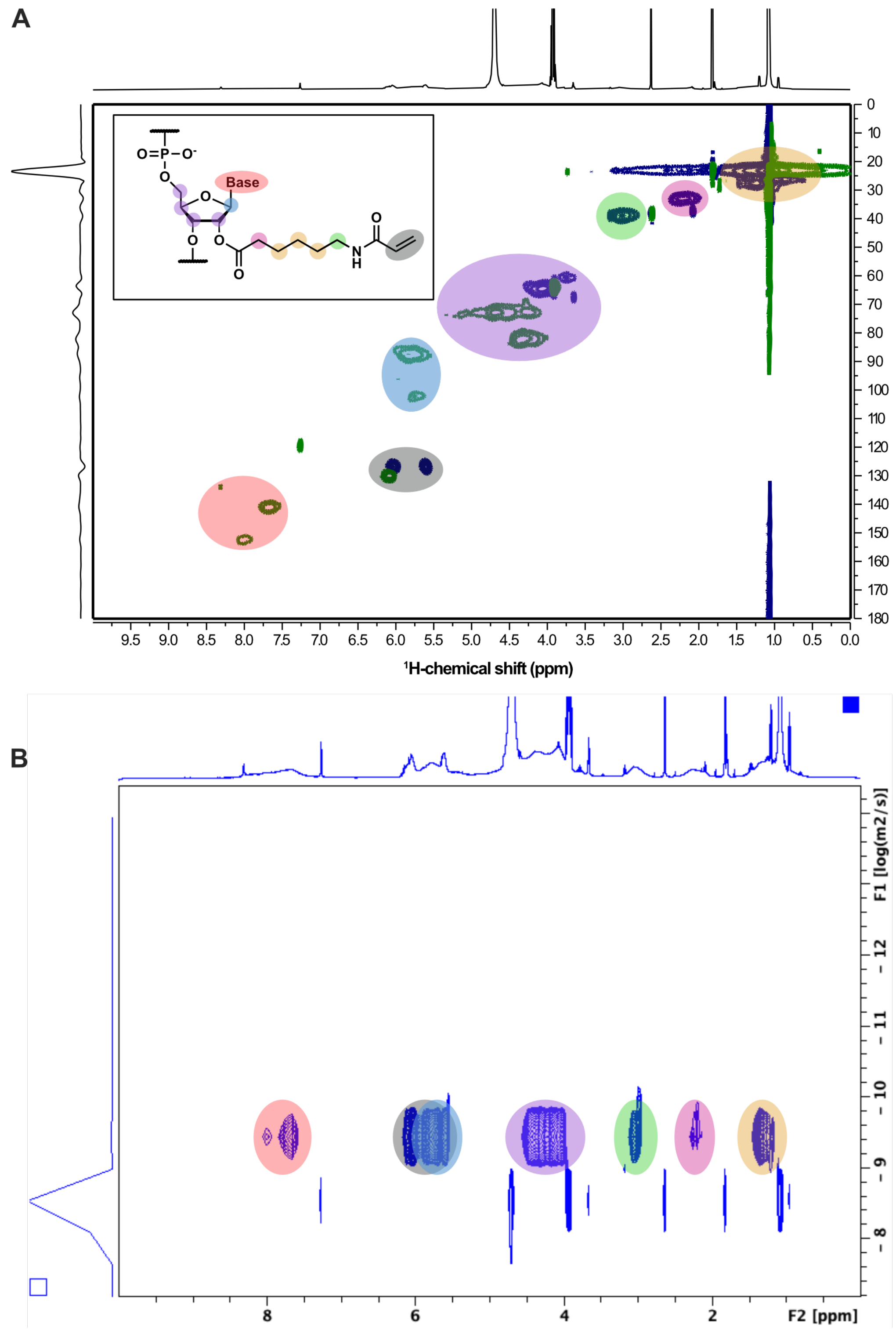

Figure S3. (A) 2D ^{1}H–^{13}C HSQC spectra of biomass RNA after the treatment of **AAm-AI** in 25% DMSO. Green peaks represent CH and blue peaks represent CH_2, respectively. (B) ^{1}H-DOSY analysis of the biomass RNA crosslinker.

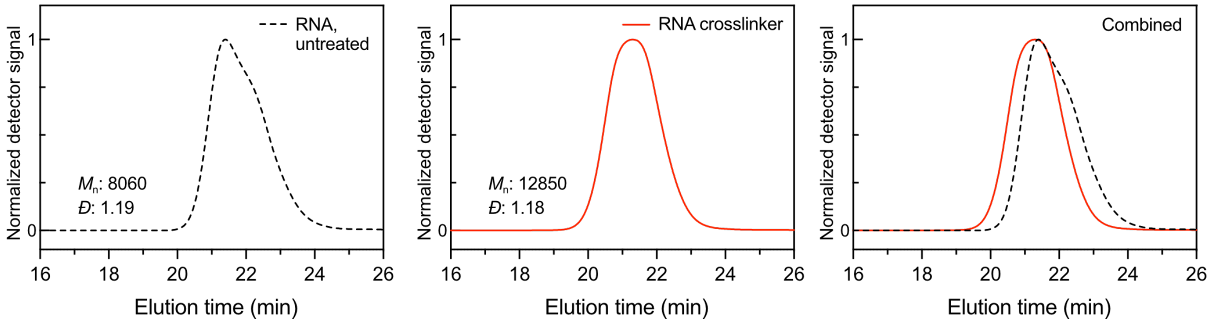

Figure S4. SEC-MALS traces of biomass RNA before and after acylation. 3 equivalents of **AAm-AI** compared to ribonucleotides were treated to RNA in 100% *v*/*v* DMSO.

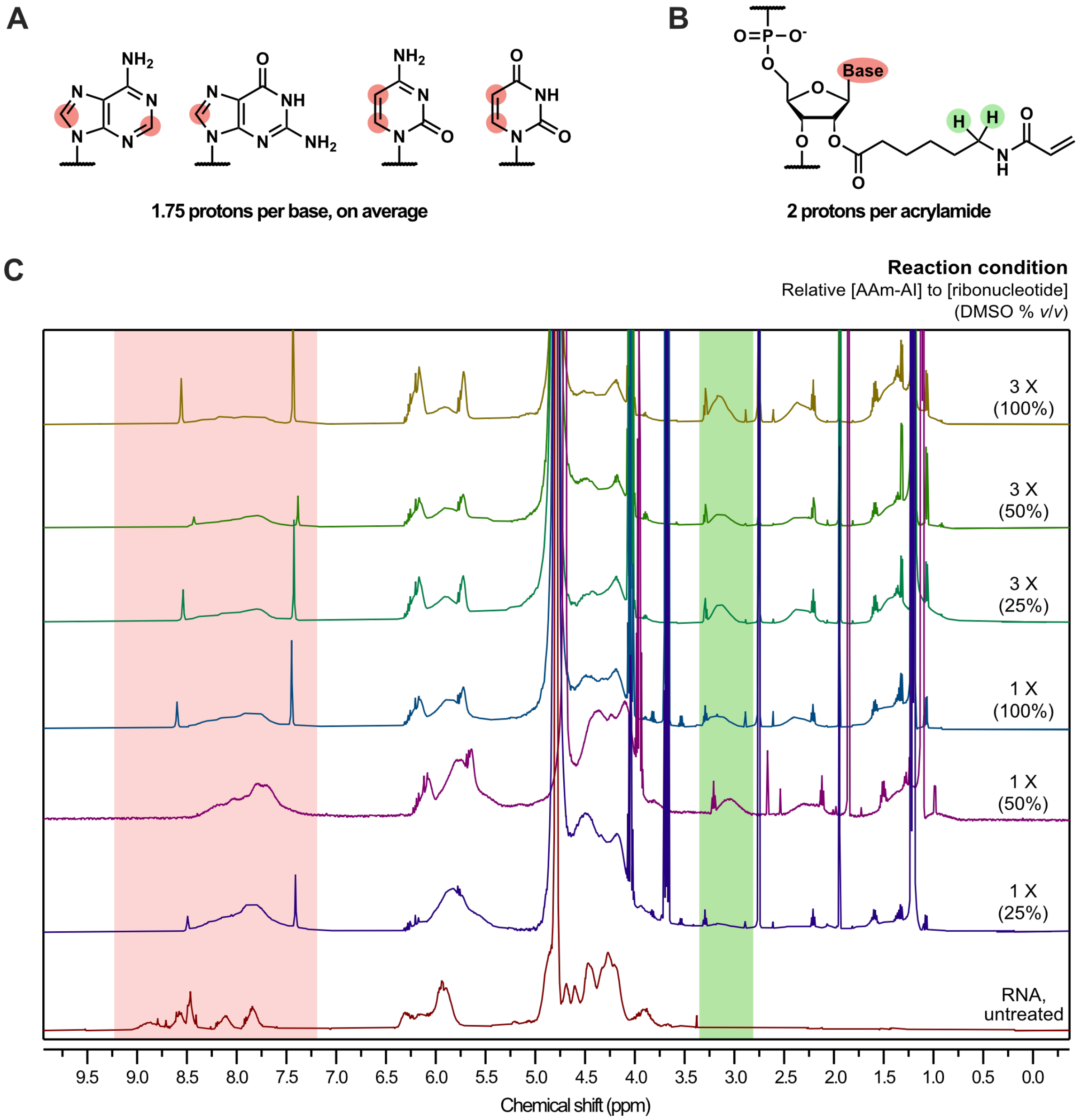

Figure S5. Determination of the degree of modification on the biomass RNA. (A) Chemical structure of the four RNA nucleobases. (B) Chemical structure of the functionalized ribonucleotide. (C) ^{1}H NMR spectra of biomass RNA after the treatment of **AAm-AI** under different reaction conditions. The red region in the range of 7.2–9.5 ppm corresponds to protons in the nucleobases (orange circles in Figures S5A and S5B). The green region in the range of 2.8–3.3 ppm corresponds to the two protons in the acrylamido residue integrated into RNA. The ratio between ribonucleotides and incorporated acrylamido residues was estimated by normalizing the area under the peak of RNA bases to 1.75, which represents the average number of protons in each RNA monomer unit. Then, the area of the NMR peak ranging from 2.8 to 3.3 ppm, which corresponds to the two protons in the acrylamido residue integrated into RNA, was obtained and halved. This resulting value provides the quantity of acrylamido modifications per ribonucleotide.

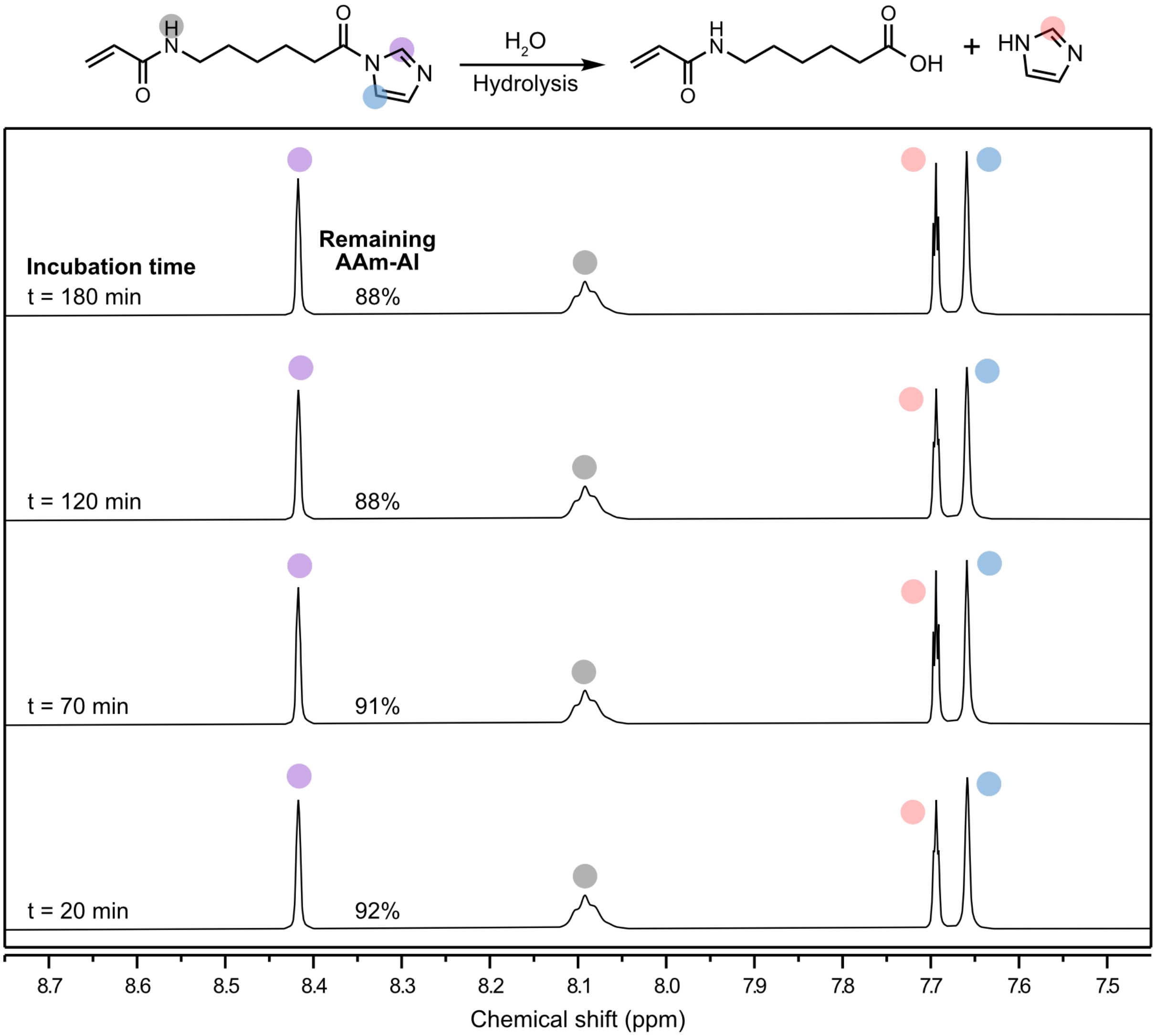

Figure S6. ^{1}H NMR spectra of **AAm-AI** in 100% DMSO-d_6 at different time intervals.

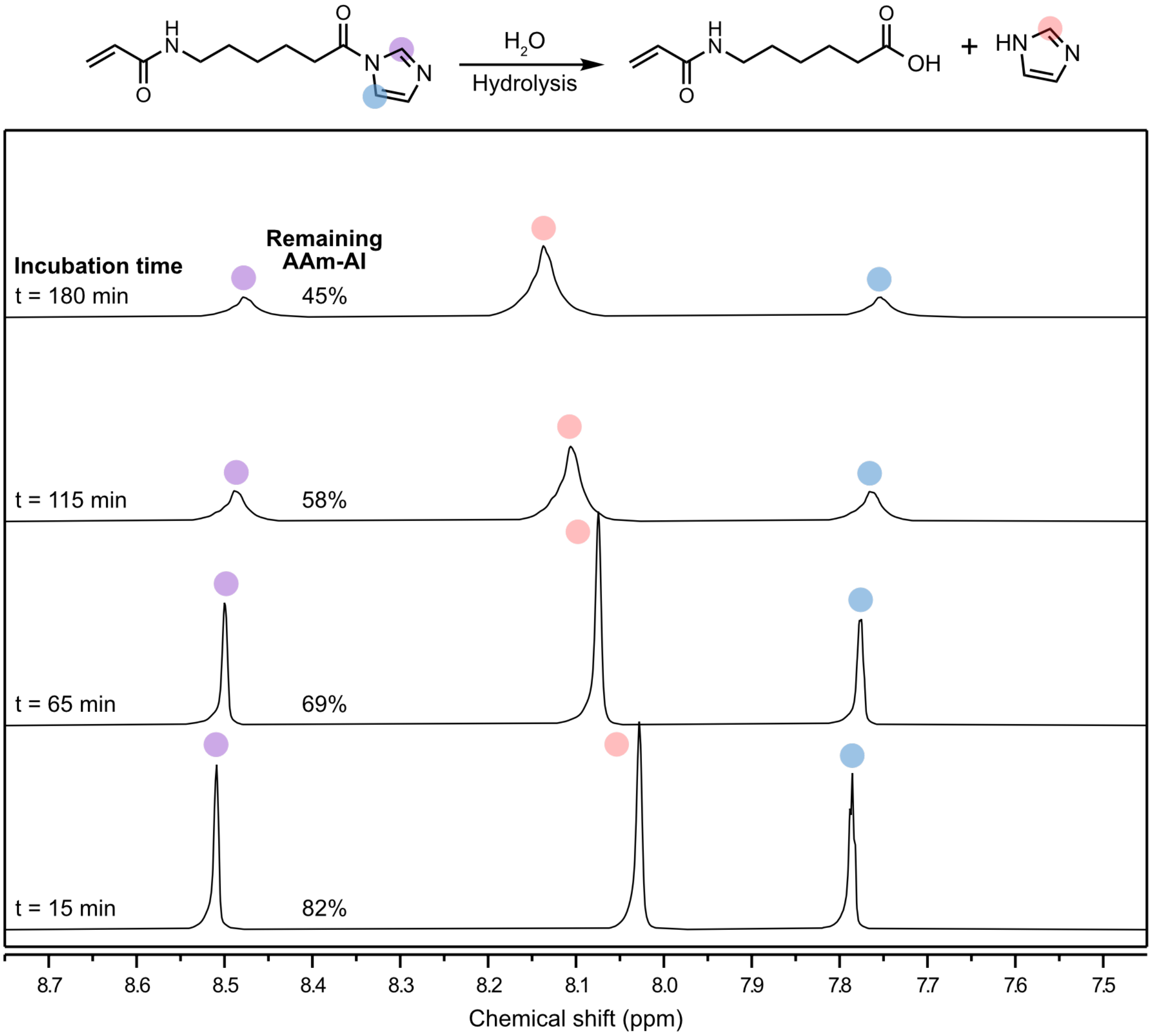

Figure S7. ^{1}H NMR spectra of **AAm-AI** in 50% DMSO-d_6 in D_2O at different time intervals.

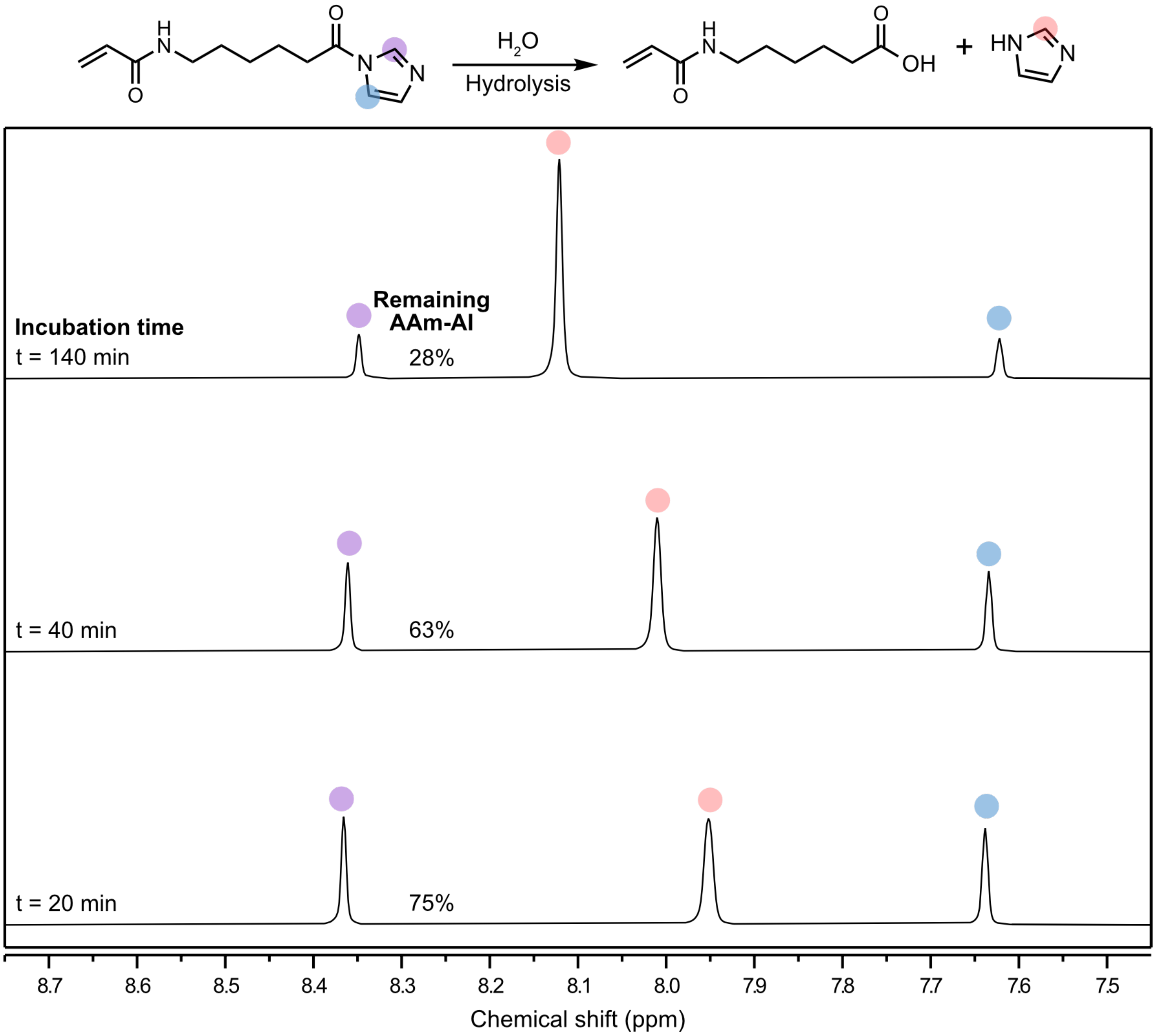

Figure S8. ^{1}H NMR spectra of **AAm-AI** in 25% DMSO-d_6 in D_2O at different time intervals.

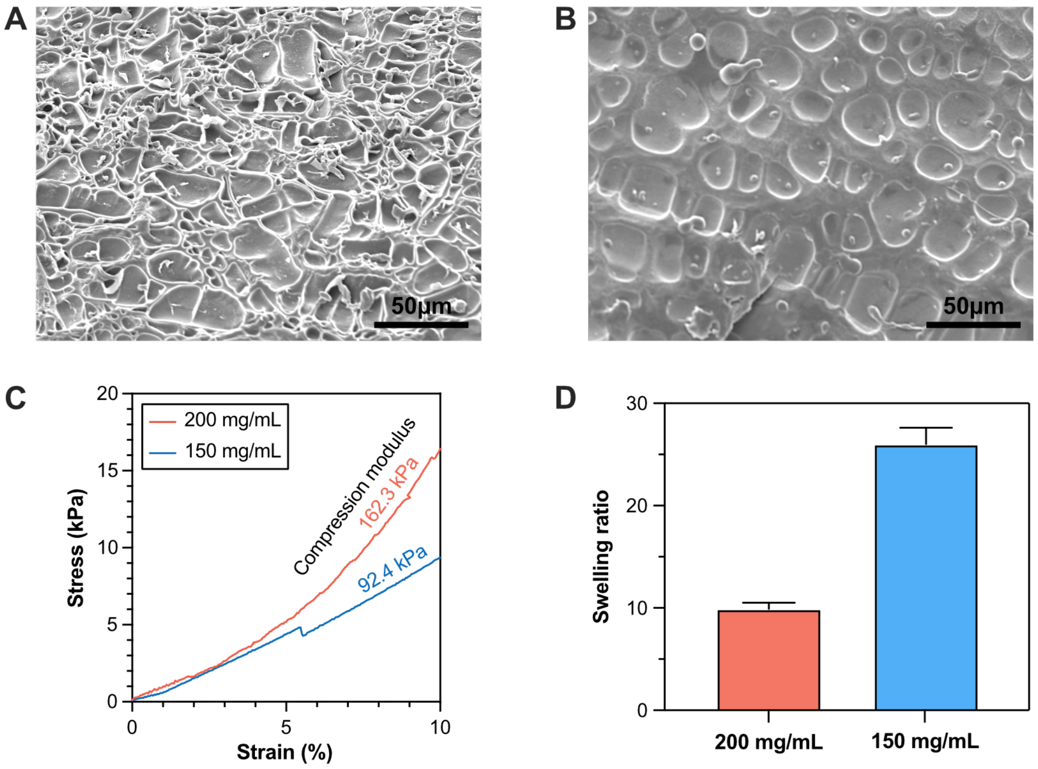

Figure S9. Comparison of RNA hydrogels (**100R$_{100}$**) with different RNA content. (A, B) SEM image of homopolymerized RNA crosslinker at the final concentration of (A) 200 mg/mL; and (B) 150 mg/mL, respectively. (C) Compressive stress-strain curve of **100R$_{100}$** with different RNA content. (D) The swelling ratio of **100R$_{100}$** with different RNA content. A summary of the reaction conditions for the synthesis of the hydrogels is shown in Table S1.

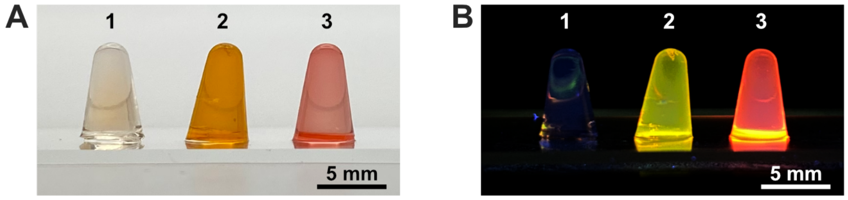

Figure S10. (A, B) Images of hydrogels after staining with nucleic acid-intercalating dyes under the (A) white light; and (B) UV light (λ = 365 nm), respectively. (**1**–**3**) 10% polyacrylamide hydrogel after the treatment of GelGreen (**1**); RNA-acrylamide hybrid gel (**$50R_{43}AAmix_{57}$**) after the treatment of GelGreen (**2**); and RNA-acrylamide hybrid gel (**$50R_{43}AAmix_{57}$**) after the treatment of GelRed (**3**), respectively. A summary of the reaction conditions for the synthesis of the hydrogels is shown in Table S2.

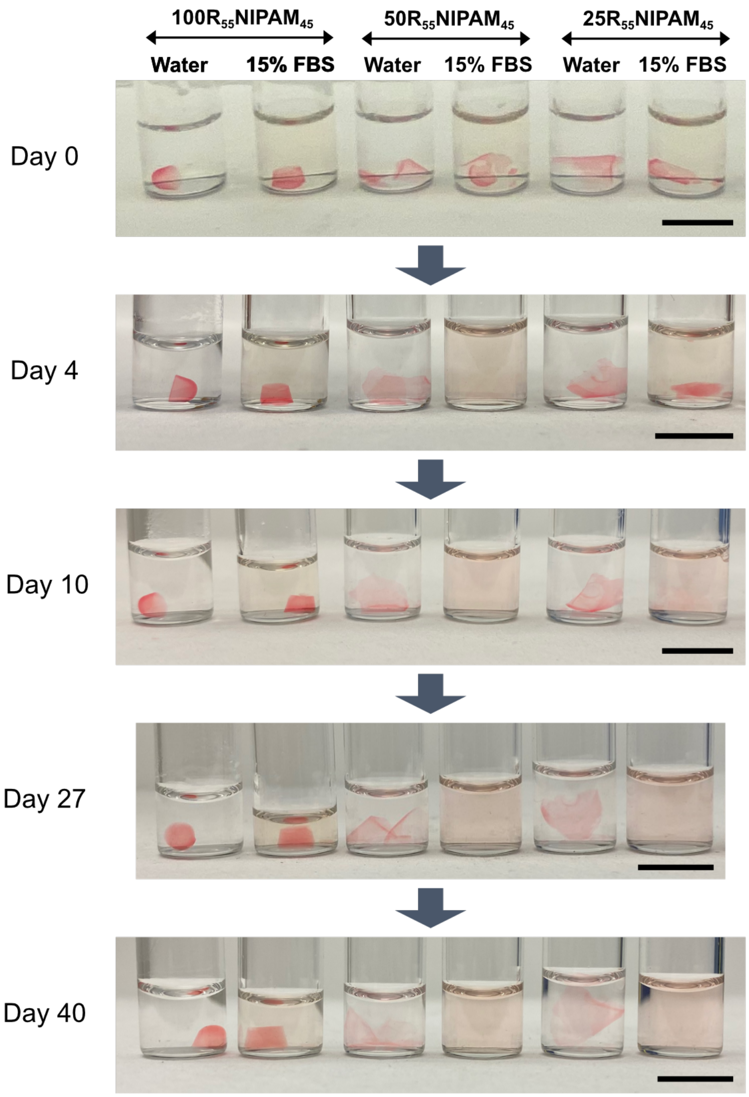

Figure S11. Degradation of RNA-NIPAM hybrid gels in 15% FBS. A summary of the reaction conditions for the synthesis of the hydrogels is shown in Table S2. The **100R50NIPAM50**, **50R50NIPAM50**, and **25R50NIPAM50** hydrogels were synthesized by copolymerization of NIPAM (50 wt%) with RNA crosslinker that was prepared under the 100%, 50%, and 25% of DMSO in water, respectively. Scale bars = 1 cm.

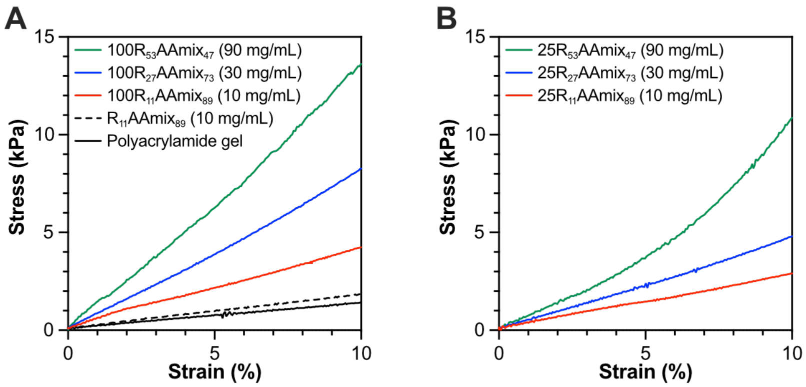

Figure S12. (A–B) Compressive stress-strain curve of RNA-acrylamide hybrid hydrogels made of RNA crosslinker synthesized under (A) 100%; and (B) 25% DMSO (*v*/*v*), respectively. **$R_{11}AAmix_{89}$** contains 10 mg/mL of unmodified RNA. A summary of the synthetic conditions for the hydrogels is shown in Table S3.

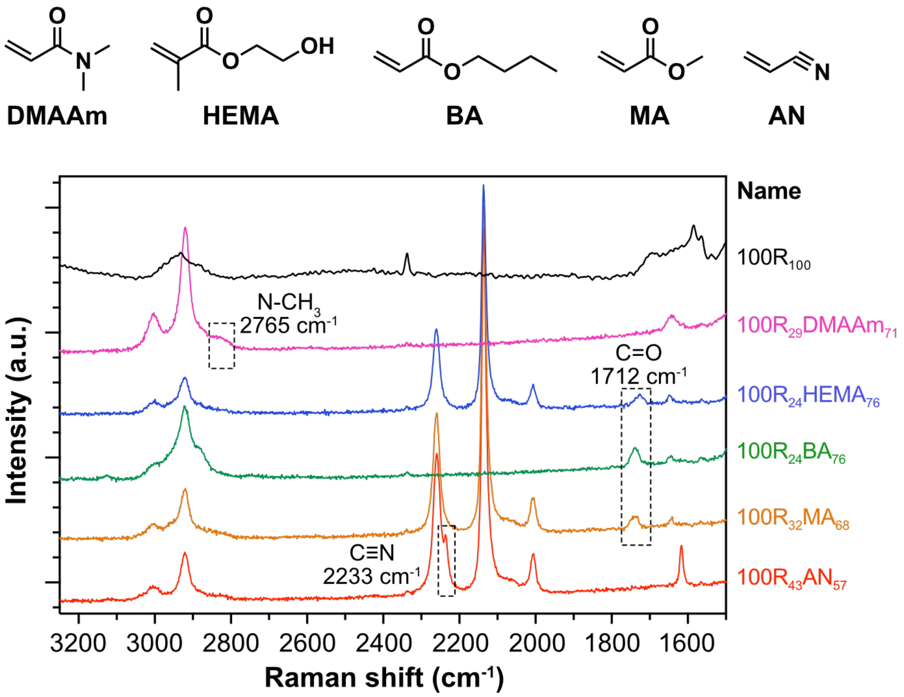

Figure S13. Raman spectra of copolymeric RNA hybrid gels made of different comonomers in DMSO. A summary of the reaction conditions is shown in Table S4. Abbreviations: DMAAm (dimethyl acrylamide); HEMA (hydroxyethyl methacrylate); BA (butyl acrylate); MA (methyl acrylate); AN (acrylonitrile).

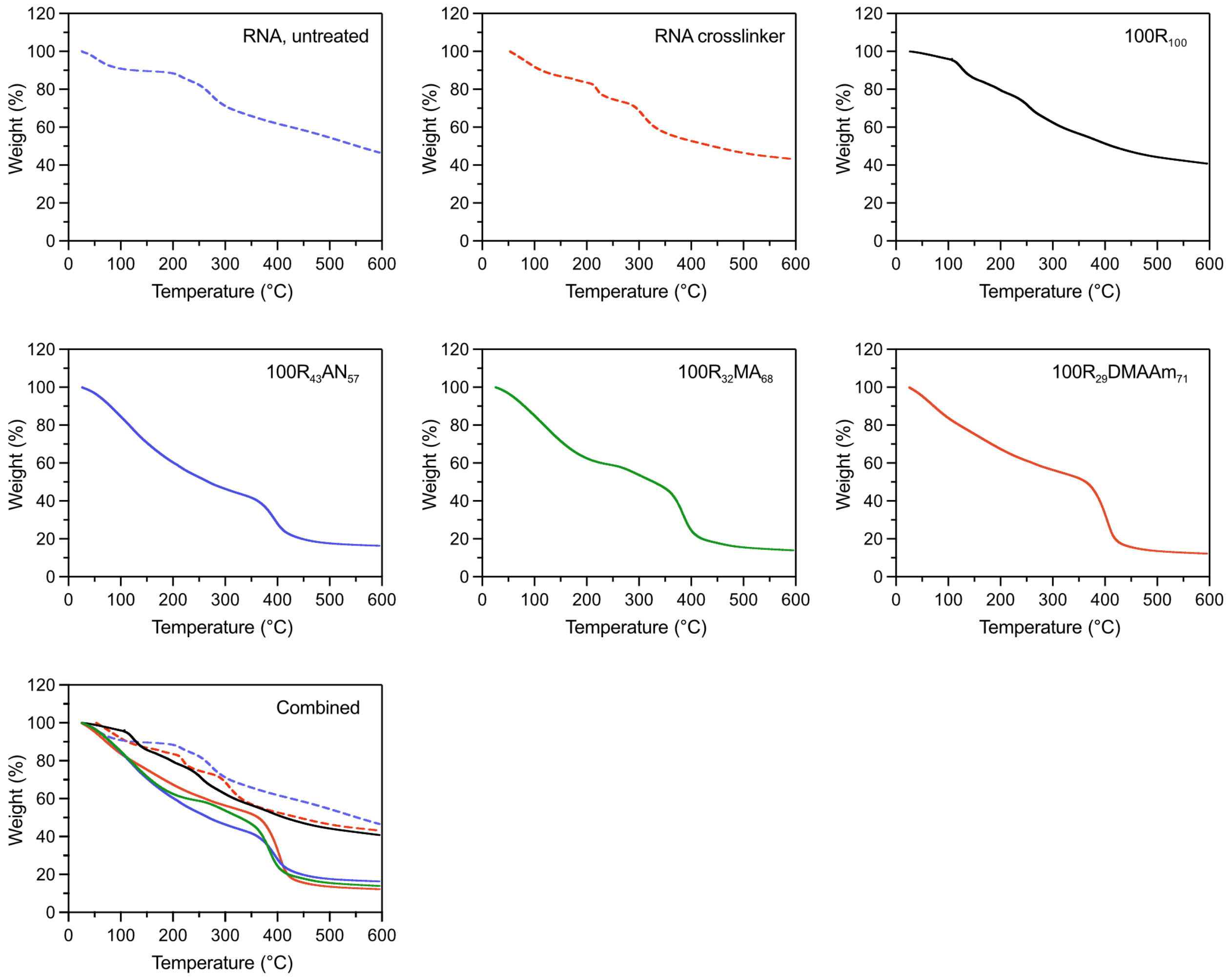

Figure S14. Thermogravimetric analysis of copolymeric RNA hydrogels made of different comonomers. Ramp: 5 °C/min from room temperature to 600 °C under nitrogen atmosphere. A summary of the reaction conditions is shown in Table S4. Abbreviations: DMAAm (dimethyl acrylamide), AN (acrylonitrile), and MA (methyl acrylate).

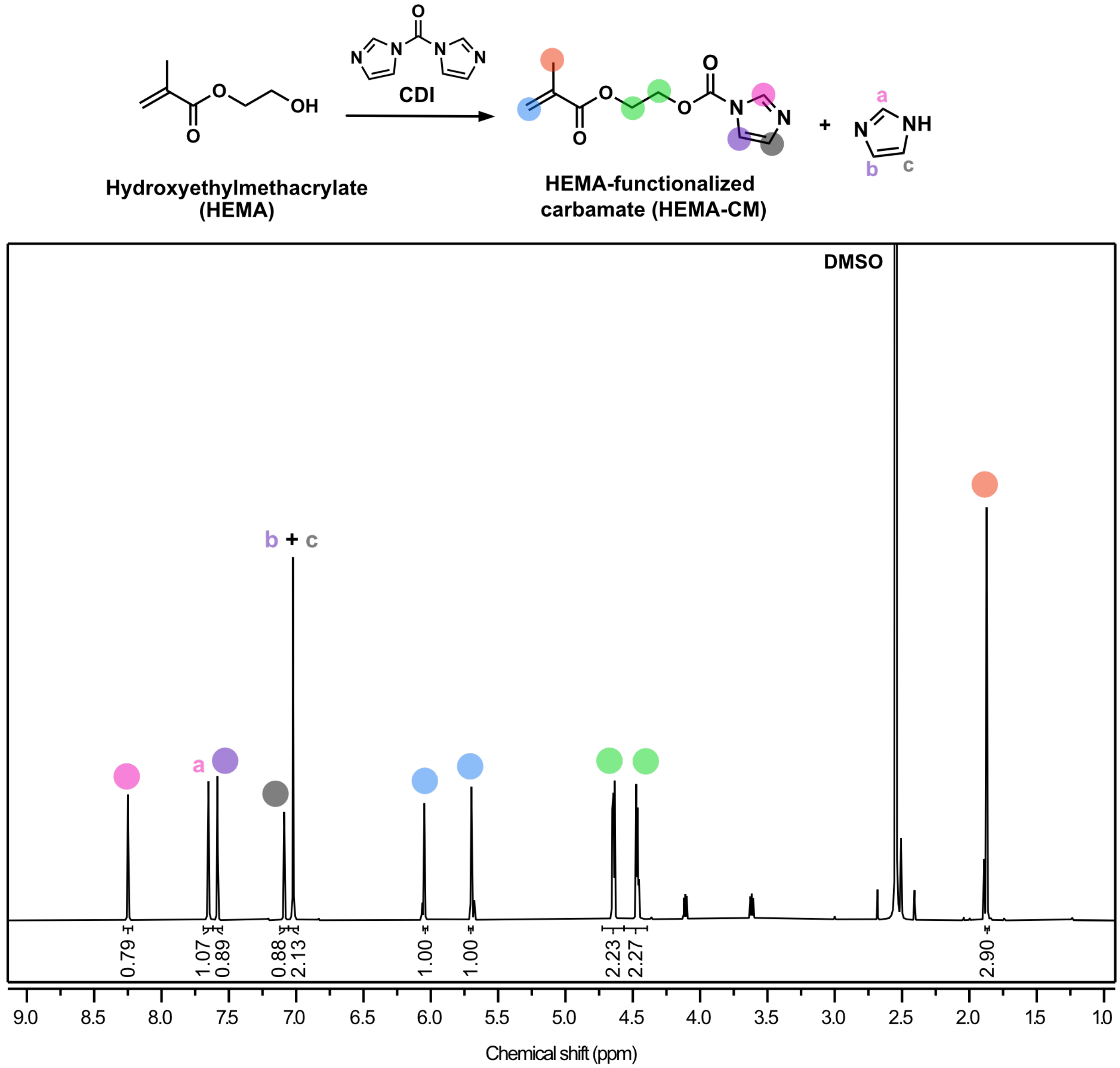

Figure S15. ^{1}H NMR spectrum of **HEMA-CM**. ^{1}H NMR (500 MHz, DMSO-d_6) δ 8.25 (t, J = 1.1 Hz, 1H), 7.65 (d, J = 1.1 Hz, 1H), 7.59 (t, J = 1.4 Hz, 1H), 7.09 (dd, J = 1.6, 0.9 Hz, 1H), 7.02 (d, J = 1.1 Hz, 2H), 6.05 (dd, J = 1.7, 1.0 Hz, 1H), 5.70 (p, J = 1.6 Hz, 1H), 4.73 – 4.56 (m, 2H), 4.56 – 4.39 (m, 2H), 1.87 (t, J = 1.3 Hz, 3H).

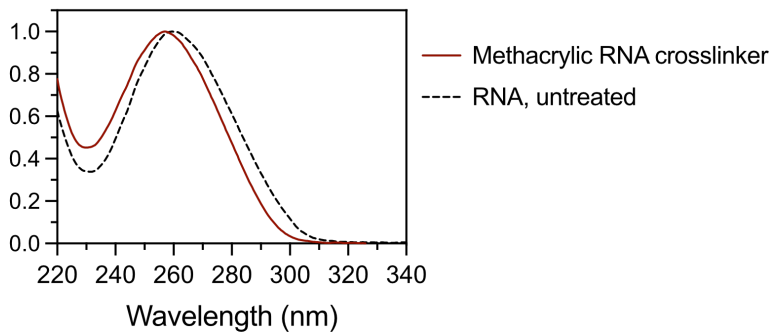

Figure S16. UV-Vis spectra of methacrylic RNA crosslinker synthesized in 50% *v*/*v* DMSO.

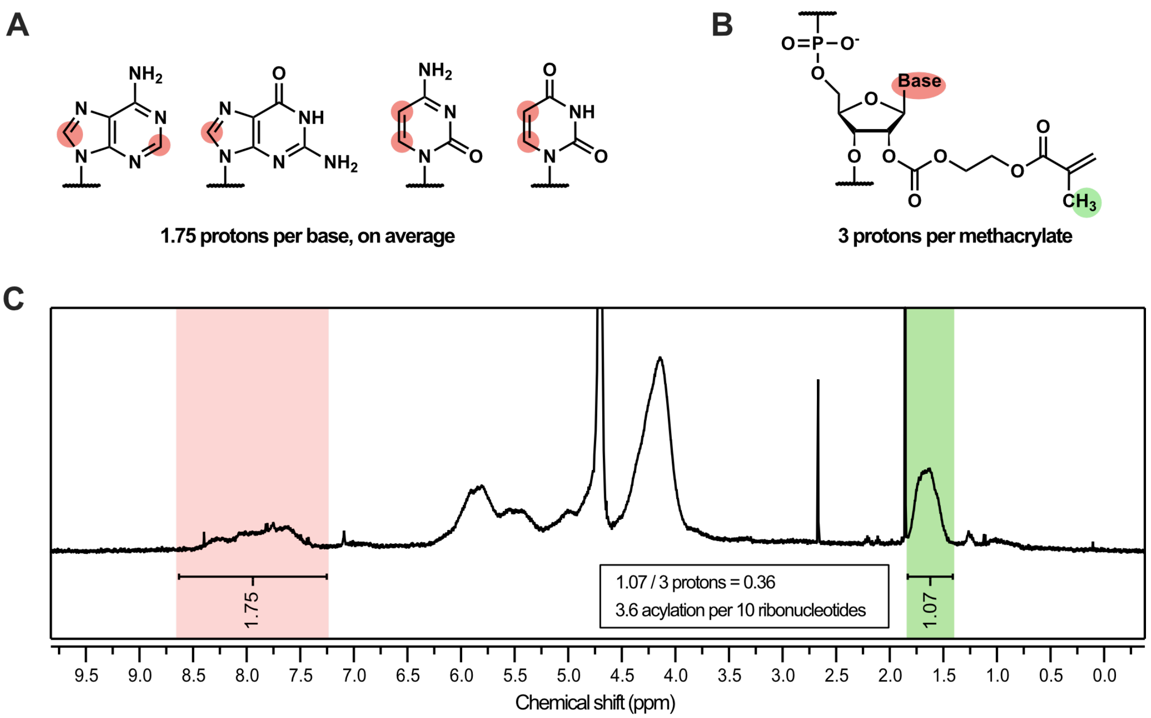

Figure S17. Determination of the degree of methacrylate modification on the biomass RNA. (A) Chemical structure of the four RNA nucleobases. (B) The chemical structure of the ribonucleotide functionalized with methacrylate residue. **c**, ^{1}H NMR spectra of biomass RNA after the treatment of **HEMA-CM** under 50 *v*/*v* % DMSO in water overnight. The red region in the range of 7.25–8.63 ppm corresponds to protons in the nucleobases (orange circles in Figures S17A and S17B). The green region in the range of 1.42–1.83 ppm corresponds to the three protons in the methacrylate residue integrated into RNA. The ratio between ribonucleotides and incorporated methacrylate residues was estimated by normalizing the area under the peak of RNA bases to 1.75, which represents the average number of protons in each RNA monomer unit. Then, the area of the NMR peak ranging from 1.42–1.83 ppm, which corresponds to the three protons in the methacrylate residue integrated into RNA, was obtained and divided by 3.

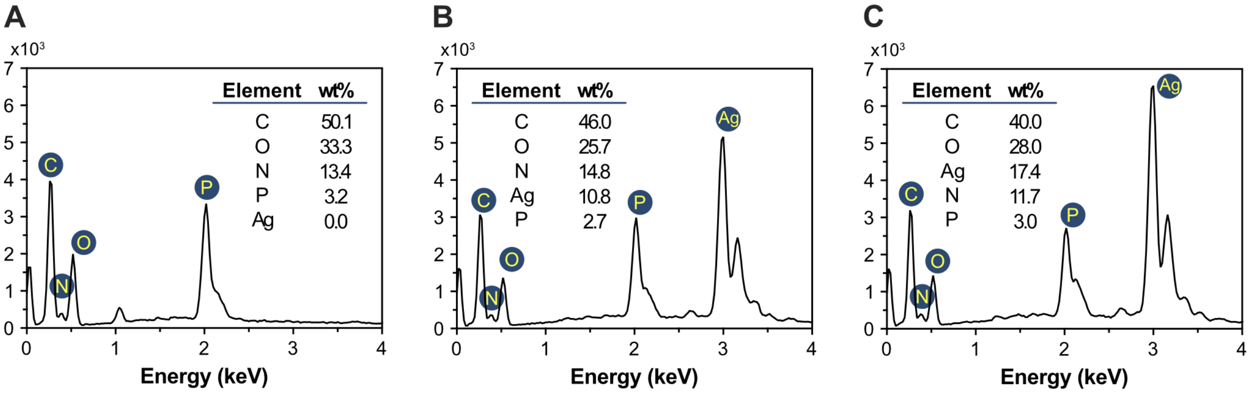

Figure S18. EDX spectra and elemental composition of hydrogels. (A) Polyacrylamide gel (**5** in Figure 6B) after incubation in 100 mM $AgNO_3$ for 3 h. (B) RNA-acrylamide hydrogel (**2** in Figure 6B) after incubation in 100 mM $AgNO_3$ for 3 h. (C) RNA-acrylamide hydrogel (**3** in Figure 6B) after incubation in 400 mM $AgNO_3$ for 3 h.

Supplementary Tables

Table S1. Summary of the reaction conditions for the synthesis of hydrogels in Figures 3 and S9.

Entry	Name	Acylation condition[a]	RNA mass (wt%)[b]	Comonomer	Volume (RNA conc.)	Method[c]
1	$100R_{100}$ (Figure 3)	100% *v*/*v* DMSO	3.75 mg (100 wt%)	-	25 µL (150 mg/mL)	FRP (APS, TEMED)
2	$50R_{100}$ (Figure 3)	50% *v*/*v* DMSO	3.75 mg (100 wt%)	-	25 µL (150 mg/mL)	FRP (APS, TEMED)
3	$25R_{100}$ (Figure 3)	25% *v*/*v* DMSO	3.75 mg (100 wt%)	-	25 µL (150 mg/mL)	FRP (APS, TEMED)
4	$100R_{100}$ (Figure S9, 200mg/mL)	100% *v*/*v* DMSO	9.00 mg (100 wt%)	-	45 µL (200 mg/mL)	FRP (APS, TEMED)
5	$100R_{100}$ (Figure S9, 150mg/mL)	100% *v*/*v* DMSO	6.75 mg (100 wt%)	-	45 µL (150 mg/mL)	FRP (APS, TEMED)

[a]3 equivalents of **AAm-AI** compared to ribonucleotides were used. [b]The weight percentage (wt%) of RNA in the gel was calculated from the following equation: (mass of RNA in the gel) / (mass of RNA + mass of comonomer) X 100. [c]Reaction was performed in water.

Table S2. Summary of the reaction conditions for the synthesis of hydrogels in Figures S10 and S11.

Entry	Name	Acylation condition[a]	RNA mass (wt%)[b]	Comonomer	Volume (RNA conc.)	Method[c]
1	Polyacrylamide gel[d] (**1** in Figure S10)	-	-	Acrylamide mix (10%)	60 µL (N/A)	FRP (APS, TEMED)
2	$50R_{43}AAmix_{57}$[d] (**2** in Figure S10)	50% *v*/*v* DMSO	4.50 mg (43 wt%)	Acrylamide mix (10%)	60 µL (75 mg/mL)	FRP (APS, TEMED)
3	$50R_{43}AAmix_{57}$[e] (**3** in Figure S10)	50% *v*/*v* DMSO	4.50 mg (43 wt%)	Acrylamide mix (10%)	60 µL (75 mg/mL)	FRP (APS, TEMED)
4	$100R_{55}NIPAM_{45}$ (Figure S11)	100% *v*/*v* DMSO	1.25 mg (55 wt%)	NIPAM (1.06 mg)	25 µL (50 mg/mL)	FRP (APS, TEMED)
5	$50R_{55}NIPAM_{45}$ (Figure S11)	50% *v*/*v* DMSO	1.25 mg (55 wt%)	NIPAM (1.06 mg)	25 µL (50 mg/mL)	FRP (APS, TEMED)
6	$25R_{55}NIPAM_{45}$ (Figure S11)	25% *v*/*v* DMSO	1.25 mg (55 wt%)	NIPAM (1.06 mg)	25 µL (50 mg/mL)	FRP (APS, TEMED)

[a]3 equivalents of **AAm-AI** compared to ribonucleotides were used. [b]The weight percentage (wt%) of RNA in the gel was calculated from the following equation: (mass of RNA in the gel) / (mass of RNA + mass of comonomer) X 100. [c]Reaction was performed in water. [d]Stained with GelGreen. [e]Stained with GelRed.

Table S3. Summary of the synthetic conditions for the hydrogels in Figures 4B–4D and S12.

Entry	Name	Acylation condition[a]	RNA mass (wt%)[b]	Comonomer	Volume (RNA conc.)	Method[c]
1	NIPAM gel[d] (**1** in Figure 4B)	-	-	NIPAM + 5% Acrylamide mix	25 µL (N/A)	FRP (APS, TEMED)
2	$25R_{55}NIPAM_{45}$ (**2** in Figure 4B)	25% *v*/*v* DMSO	1.25 mg (55 wt%)	NIPAM (1.06 mg)	25 µL (50 mg/mL)	FRP (APS, TEMED)
3	$50R_{55}NIPAM_{45}$ (**3** in Figure 4B)	50% *v*/*v* DMSO	1.25 mg (55 wt%)	NIPAM (1.06 mg)	25 µL (50 mg/mL)	FRP (APS, TEMED)
4	$100R_{55}NIPAM_{45}$ (**4** in Figure 4B)	100% *v*/*v* DMSO	1.25 mg (55 wt%)	NIPAM (1.06 mg)	25 µL (50 mg/mL)	FRP (APS, TEMED)
5	$25R_{67}AAmix_{33}$ (Figure 4C)	25% *v*/*v* DMSO	32 mg (67 wt%)	Acrylamide mix (2%)	800 µL (40 mg/mL)	FRP (APS, TEMED)
6	$100R_{53}AAmix_{47}$ (Figures 4D, S12A)	100% *v*/*v* DMSO	4.1 mg (53 wt%)	Acrylamide mix (8%)	45 µL (90 mg/mL)	FRP (APS, TEMED)
7	$100R_{27}AAmix_{73}$ (Figures 4D, S12A)	100% *v*/*v* DMSO	1.4 mg (27 wt%)	Acrylamide mix (8%)	45 µL (30 mg/mL)	FRP (APS, TEMED)
8	$100R_{11}AAmix_{89}$ (Figures 4D, S12A)	100% *v*/*v* DMSO	0.5 mg (11 wt%)	Acrylamide mix (8%)	45 µL (10 mg/mL)	FRP (APS, TEMED)
9	$R_{11}AAmix_{89}$[e] (Figure S12A)	-	0.5 mg (11 wt%)	Acrylamide mix (8%)	45 µL (10 mg/mL)	FRP (APS, TEMED)
10	Polyacrylamide gel (Figure S12A)	-	-	Acrylamide mix (8%)	45 µL (N/A)	FRP (APS, TEMED)
11	$25R_{53}AAmix_{47}$ (Figures 4D, S12B)	25% *v*/*v* DMSO	4.1 mg (53 wt%)	Acrylamide mix (8%)	45 µL (90 mg/mL)	FRP (APS, TEMED)
12	$25R_{27}AAmix_{73}$ (Figures 4D, S12B)	25% *v*/*v* DMSO	1.4 mg (27 wt%)	Acrylamide mix (8%)	45 µL (30 mg/mL)	FRP (APS, TEMED)
13	$25R_{11}AAmix_{89}$ (Figures 4D, S12B)	25% *v*/*v* DMSO	0.5 mg (11 wt%)	Acrylamide mix (8%)	45 µL (10 mg/mL)	FRP (APS, TEMED)

[a]3 equivalents of **AAm-AI** compared to ribonucleotides were used. [b]The weight percentage (wt%) of RNA in the gel was calculated from the following equation: (mass of RNA in the gel) / (mass of RNA + mass of comonomer) X 100. [c]Reaction was performed in water. [d]5% acrylamide mix was added to 1.06 mg of NIPAM as the crosslinker. [e]Biomass RNA without the treatment of **AAm-AI** was used.

Table S4. Summary of the synthetic conditions for the hydrogels in Figures 4E, 4F, S13, and S14.

Entry	Name	Acylation condition[a]	RNA mass (wt%)[b]	Comonomer	Volume (RNA conc.)	Method[c]
1	MA (Figure 4E, left)	-	-	MA (103 mg)	800 µL (N/A)	FRP (Irgacure 2959)
2	MA + RNA[d] (Figure 4E, middle)	-	48 mg (32 wt%)	MA (103 mg)	800 µL (60 mg/mL)	FRP (Irgacure 2959)
3	$100R_{32}MA_{68}$ (Figure 4E, right)	100% *v*/*v* DMSO	48 mg (32 wt%)	MA (103 mg)	800 µL (60 mg/mL)	FRP (Irgacure 2959)
4	$100R_{29}DMAAm_{71}$	100% *v*/*v* DMSO	48 mg (29 wt%)	DMAAm (156 mg)	800 µL (60 mg/mL)	FRP (Irgacure 2959)
5	$100R_{24}HEMA_{76}$	100% *v*/*v* DMSO	48 mg (24 wt%)	HEMA (156 mg)	800 µL (60 mg/mL)	FRP (Irgacure 2959)
6	$100R_{24}BA_{76}$	100% *v*/*v* DMSO	48 mg (24 wt%)	BA (154 mg)	800 µL (60 mg/mL)	FRP (Irgacure 2959)
7	$100R_{43}AN_{57}$	100% *v*/*v* DMSO	48 mg (43 wt%)	AN (64 mg)	800 µL (60 mg/mL)	FRP (Irgacure 2959)
8	$100R_{100}$	100% *v*/*v* DMSO	120 mg (100 wt%)	-	800 µL (60 mg/mL)	FRP[e] (APS, TEMED)

[a]3 equivalents of **AAm-AI** compared to ribonucleotides were used. [b]The weight percentage (wt%) of RNA in the gel was calculated from the following equation: (mass of RNA in the gel) / (mass of RNA + mass of comonomer) X 100. [c]Reaction was performed in DMSO under the irradiation of UV light (λ = 365 nm). [d]Biomass RNA without the treatment of **AAm-AI** was used. [e]Reaction was performed in water. Abbreviations: DMAAm (dimethyl acrylamide), HEMA (hydroxyethyl methacrylate), BA (butyl acrylate), MA (methyl acrylate), and AN (acrylonitrile).

Table S5. Summary of the reaction conditions for the synthesis of hydrogels in Figure 5.

Entry	Name	Acylation condition[a]	RNA mass (wt%)[b]	Comonomer	Volume (RNA conc.)	Method[c]
1	$50R_{27}OEOMA_{73}$ (Figure 5B)	50% *v/v* DMSO	75 mg (27 wt%)	$OEOMA_{500}$ (200 mg)	1mL (75 mg/mL)	PET-RAFT
2	$50R_{27}OEOMA_{73}$ (Figure 5C)	50% *v/v* DMSO	75 mg (27 wt%)	$OEOMA_{500}$ (200 mg)	1mL (75 mg/mL)	EY-ATRP
3	$50R_{16}OEOMA_{84}$ (Figure 5D)	50% *v/v* DMSO	36 mg (15 wt%)	$OEOMA_{500}$ (200 mg)	1mL (37 mg/mL)	PET-RAFT
4	$50R_{16}OEOMA_{84}$ (Figure 5D)	50% *v/v* DMSO	36 mg (15 wt%)	$OEOMA_{500}$ (200 mg)	1mL (37 mg/mL)	EY-ATRP
5	$50R_{16}OEOMA_{84}$ (Figure 5D)	50% *v/v* DMSO	36 mg (15 wt%)	$OEOMA_{500}$ (200 mg)	1mL (37 mg/mL)	FRP (Irgacure 2959)

[a]6 equivalents of **HEMA-CM** compared to ribonucleotides were used. [b]The weight percentage (wt%) of RNA in the gel was calculated from the following equation: (mass of RNA in the gel) / (mass of RNA + mass of comonomer) X 100. [c]Reaction was performed in 1X PBS under the irradiation of green light (λ = 540 nm, for PET-RAFT or EY-ATRP) or UV light (λ = 365 nm, for FRP), respectively.

Table S6. Summary of the reaction conditions for the synthesis of hydrogels in Figure 6.

Entry	Name	Acylation condition[a]	RNA mass (wt%)[b]	Volume (RNA conc.)	Post-synthetic treatment[c]
1	$50R_{50}AAmix_{50}$ (**1** in Figure 6B)	50% *v*/*v* DMSO	8 mg (50 wt%)	100 µL (80 mg/mL)	water (3 h)
2	$50R_{50}AAmix_{50}$ (**2** in Figure 6B)	50% *v*/*v* DMSO	8 mg (50 wt%)	100 µL (80 mg/mL)	100 mM $AgNO_3$ in water (3 h)
3	$50R_{50}AAmix_{50}$ (**3** in Figure 6B)	50% *v*/*v* DMSO	8 mg (50 wt%)	100 µL (80 mg/mL)	400 mM $AgNO_3$ in water (3 h)
4	Polyacrylamide gel (**4** in Figure 6B)	-	-	100 µL (N/A)	water (3 h)
5	Polyacrylamide gel (**5** in Figure 6B)	-	-	100 µL (N/A)	100 mM $AgNO_3$ in water (3 h)
6	Polyacrylamide gel (**6** in Figure 6B)	-	-	100 µL (N/A)	400 mM $AgNO_3$ in water (3 h)
7	$50R_{50}AAmix_{50}$ (Figures 6D, 6E)	50% *v*/*v* DMSO	17.6 mg (50 wt%)	220 µL (80 mg/mL)	100 mM $AgNO_3$ in water (overnight)
8	$50R_{33}AAmix_{67}$ (Figures 6D, 6E)	50% *v*/*v* DMSO	8.8 mg (33 wt%)	220 µL (40 mg/mL)	100 mM $AgNO_3$ in water (overnight)
9	$50R_{20}AAmix_{80}$ (Figures 6D, 6E)	50% *v*/*v* DMSO	4.4 mg (20 wt%)	220 µL (20 mg/mL)	100 mM $AgNO_3$ in water (overnight)
10	$50R_{11}AAmix_{89}$ (Figures 6D, 6E)	50% *v*/*v* DMSO	2.2 mg (11 wt%)	220 µL (10 mg/mL)	100 mM $AgNO_3$ in water (overnight)
11	$50R_{6}AAmix_{94}$ (Figures 6D, 6E)	50% *v*/*v* DMSO	1.1 mg (6 wt%)	220 µL (5 mg/mL)	100 mM $AgNO_3$ in water (overnight)
12	Polyacrylamide gel (Figures 6D, 6E)	-	-	220 µL (N/A)	100 mM $AgNO_3$ in water (overnight)
13	$50R_{33}AAmix_{67}$ (Figure 6D)	50% *v*/*v* DMSO	8.8 mg (33 wt%)	220 µL (40 mg/mL)	water (overnight)
14	Polyacrylamide gel (Figure 6D)	-	-	220 µL (N/A)	water (overnight)

All gels were synthesized by free radical polymerization in water using APS and TEMED using acrylamide mix (final concentration of 8%) as the (co)monomer. [a]3 equivalents of **AAm-AI** compared to ribonucleotides were used. [b]The weight percentage (wt%) of RNA in the gel was calculated from the following equation: (mass of RNA in the gel) / (mass of RNA + mass of comonomer).
